# Uncovering temperature-dependent exciton-polariton relaxation mechanisms in hybrid organic-inorganic perovskites

**DOI:** 10.1038/s41467-023-37772-7

**Published:** 2023-04-27

**Authors:** Madeleine Laitz, Alexander E. K. Kaplan, Jude Deschamps, Ulugbek Barotov, Andrew H. Proppe, Inés García-Benito, Anna Osherov, Giulia Grancini, Dane W. deQuilettes, Keith A. Nelson, Moungi G. Bawendi, Vladimir Bulović

**Affiliations:** 1 Department of Electrical Engineering and Computer Science, Massachusetts Institute of Technology, Cambridge, MA USA; 2 Department of Chemistry, Massachusetts Institute of Technology, Cambridge, MA USA; 3 Department of Organic Chemistry, Universidad Complutense de Madrid, Madrid, Spain; 4 Department of Chemistry & INSTM, University of Pavia, Pavia, Italy; 5 Research Laboratory of Electronics, Massachusetts Institute of Technology, Cambridge, MA USA

**Keywords:** Nanocavities, Two-dimensional materials, Polaritons, Ultrafast photonics, Electronic properties and materials

## Abstract

Hybrid perovskites have emerged as a promising material candidate for exciton-polariton (polariton) optoelectronics. Thermodynamically, low-threshold Bose-Einstein condensation requires efficient scattering to the polariton energy dispersion minimum, and many applications demand precise control of polariton interactions. Thus far, the primary mechanisms by which polaritons relax in perovskites remains unclear. In this work, we perform temperature-dependent measurements of polaritons in low-dimensional perovskite wedged microcavities achieving a Rabi splitting of ${{{\hslash }}\Omega }_{{Rabi}}$ = 260 ± 5 meV. We change the Hopfield coefficients by moving the optical excitation along the cavity wedge and thus tune the strength of the primary polariton relaxation mechanisms in this material. We observe the polariton bottleneck regime and show that it can be overcome by harnessing the interplay between the different excitonic species whose corresponding dynamics are modified by strong coupling. This work provides an understanding of polariton relaxation in perovskites benefiting from efficient, material-specific relaxation pathways and intracavity pumping schemes from thermally brightened excitonic species.

## Abstract (web summary)

Exciton-polaritons present opportunities for quantum photonics, next generation qubits, and tuning material photophysics. Here Laitz et al. study the temperature dependence of 2D perovskite microcavity polaritons, revealing material-specific relaxation mechanisms towards the control of polariton momentum.

## Introduction

Exciton-polaritons (polaritons) are formed in optical microcavities in the strong coupling regime between material excitons and cavity photons. This quantum superposition results in a hybrid state of light and matter, and the formation of a bosonic quasi-particle^1^. Polaritons can be modified to adjust the photonic/excitonic character, so that, even when tuned to have a large photonic fraction, polaritons can interact due to the non-zero excitonic component. Such interactions provide several advantages over purely photonic systems in designing logic elements in integrated circuits, such as facile cascadability^2^ and large nonlinearities due to the polariton matter component^3^, creating the potential for engineering fast, low-power optical transistors^4^. These properties also establish opportunities for studying Bose-Einstein condensation^5^, quantum vortices^6^, and low-threshold polariton lasing^7^ for quantum photonic technologies^8^ and next-generation qubits^9^. Additionally, recent reports utilizing strong coupling to modify electronic structure and energy transfer rates show great promise for the polariton-mediated tuning of chemical reactivity and photophysics^10^. The possibilty of improving energy conversion processes without synthetic changes to the chemical system allows for external modification of kinetics, energy, and electronic and vibrational transitions^11,12^ by the precise engineering and control of strongly coupled device structures^13^.

To date, polaritons have been sustained in inorganic materials (e.g., GaN, ZnO)^14^, organics (e.g., J-aggregates)^15^, transition metal dichalcogenides (e.g., WS_2_, WSe_2_)^16^, and perovskites (e.g., CsPbBr_3_, CsPbCl_3_, (C_6_H_5_(CH_2_)_2_NH_3_)_2_PbI_4_)^17^. In perovskites, room-temperature polariton formation^17^, manipulation^18^, lasing^19^, and condensation^20^ have been shown, in which perovskite single crystals, exfoliated flakes, platelets, or thin films are embedded in microcavities typically comprised of either a single distributed Bragg reflector (DBR) and metallic mirror or two high-quality DBRs. Although these demonstrations have shown the exceptional potential of perovskite materials for polaritonics, most optoelectronic applications rely on careful control of polariton momentum. Currently, for perovskites, the scattering and relaxation mechanisms that lead to changes in polariton energy and momentum are not well understood. Additionally, there have been very few demonstrations of polariton lasing and condensation in quasi-two-dimensional (herein referred to as 2D) perovskites, which is believed to result from the low room-temperature photoluminescence quantum efficiency (PLQE), low quality factor (Q) cavities leading to short polariton lifetimes, and high exciton-exciton annihilation rates^17,21^. Therefore, a deeper understanding of polariton scattering and relaxation mechanisms is needed in order to fully harness polariton utility for a wide range of applications.

Despite these challenges, 2D perovskites remain one of the most promising materials for room-temperature polaritonic devices due to optimal optical properties, chemical versatility, and facile deposition and fabrication schemes. 2D perovskites function as self-assembled quantum well structures, and can be formed as single crystals or polycrystalline thin films. The smaller band gap inorganic monolayers (e.g., PbI_4_), where the excitons are confined, act as the quantum well and the larger energy gap organic spacers serve as potential barriers^22^. The low Stokes shift, high absorption coefficient, narrow emission, controllable dipole orientation^23^, and high exciton binding energy (100–500 meV)^24^ render these materials excellent candidates for facilely fabricated polaritonic devices^17^.

Here we explore temperature-dependent polariton formation and relaxation in a test-bed 2D system based on phenethylammonium lead iodide perovskite (C_6_H_5_(CH_2_)_2_NH_3_)_2_PbI_4_ (PEA_2_PbI_4_) thin films embedded in a wedged microcavity and demonstrate, to the best of our knowledge, a record room-temperature Rabi splitting for PEA_2_PbI_4_ of ${{{\hslash }}\Omega }_{{Rabi}}$ = 260 ± 5 meV. This figure of merit denotes the magnitude of the photon-exciton coupling, and larger Rabi splittings lead to reduced BEC thresholds as demonstrated by nonequilibrium models^25^ and experimental work in organic polaritons^26^. We probe polariton formation by Fourier spectroscopy to image momentum (in-plane *k*_*||*_) space via reflectivity and photoluminescence (PL) measurements. We show the emergence of a polariton bottleneck for negative cavity detunings (${E}_{{cav}}-{E}_{{exc}}\, < \,0$), where *E*_*cav*_ is the energy of the microcavity photon and *E*_*exc*_ is the energy of the perovskite thin film exciton, and explore temperature-dependent polariton photophysics, revealing the role of and interplay between reservoir excitons, phonons, and cavity polaritons for efficient polariton relaxation in these materials. We measure the significant brightening of the dark excitons in PEA_2_PbI_4_ perovskite polariton structures at cryogenic temperatures, and suggest their role together with the role of biexcitons and resonant longitudinal optical (LO) phonon interactions as they jointly mediate exciton-polariton relaxation to *k*_*||*_ = 0. Additionally, we show that perovskite excited states can be tuned via strong coupling to achieve new dynamics (i.e., kinetic rates). These results yield new insights on strong coupling mechanisms in low-dimensional perovskites, informing microcavity and polariton dispersion design to harness material-specific, efficient polariton relaxation pathways to *k*_*||*_ = 0.

## Results and discussion

### Room-temperature strong coupling in 2D perovskite microcavities

**Fig. 1:**
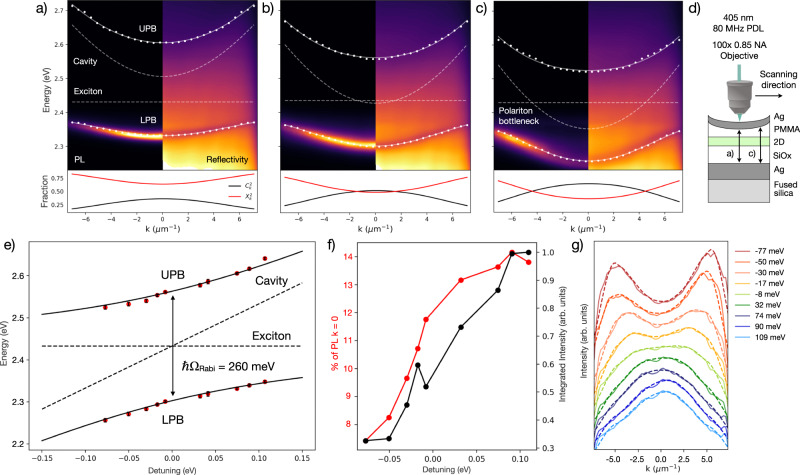
Room-temperature exciton-polariton formation. Exciton-polariton photoluminescence (PL) (left) and reflectivity (right) dispersions with increasing cavity length from **a** higher cavity mode energy to **b**, **c** lower cavity mode energy as shown schematically in **d**. As the cavity shifts to lower energies and the polariton dispersion becomes increasingly photonic **c**, the bottleneck effect emerges with the greatest emission intensity at high *k*_*||*_ values. UPB and LPB energies are extracted from reflectivity minima (white dotted line) and fitted (white solid line) using Eq. 1 (fitted cavity and exciton energies shown, white dashed line) with a Rabi splitting of ${{{\hslash }}\Omega }_{{Rabi}}$ = 260 meV. (**a**–**c**, lower figures) Hopfield coefficients for cavity detunings (photonic fraction C_k_^2^, black trace; excitonic fraction X_k_^2^, red trace) ranging from **a** excitonic to **c** photonic depicting the light-matter characteristics of the generated polaritons as a function of *k*_*||*_ (Eq. 2). **e** Experimental (red dots) and theoretical (black traces) upper and lower polariton branch energies at *k*_*||*_ = 0 with ${{{\hslash }}\Omega }_{{Rabi}}$ = 260 meV. Dashed black traces correspond to the exciton energy and cavity energy changing with cavity length. Fit error as determined by RSS. **f** As the cavity energy decreases and the dispersion becomes more negatively detuned, the photoluminescence (PL) distribution shifts to higher *k*_*||*_, resulting in a decrease in the fraction of PL within *k*_*||*_ = 0 ± 0.2 µm^−1^ (red trace). Integrated PL intensity as a function of increasingly negative detuning exhibits inhibited emission (black trace). **g** The energy-integrated PL spectra reveal the re-distribution of the maximum PL intensity to higher *k*_*||*_ with increasingly photonic detunings (raw data, solid trace; smoothed data, dashed trace).

We realize room-temperature polaritons by fabricating $\lambda /2$ metallic microcavities with a spin-cast PEA_2_PbI_4_ active layer posessing a high degree of crystallinity resembling single crystals (Fig. S1)^27^. The strongly coupled system achieves a Rabi splitting of ${{{\hslash }}\Omega }_{{Rabi}}$ = 260 meV, which is, to the best of our knowledge, the highest reported coupling strength in a PEA_2_PbI_4_ planar microcavity^17^. High-quality epitaxially-grown GaAs quantum well microcavities have previously been fabricated in a wedged geometry, which yields a spatially-varying cavity length that allows for the probing of multiple cavity detunings to investigate polaritons with varying photonic/excitonic character^28^. The reflectivity and photoluminescence dispersions are probed by Fourier spectroscopy, in which the emission of the lower polariton branch (LPB) is observed in photoluminescence and both the upper polariton branch (UPB) and LPB can be resolved in reflectivity (Figs. 1a–c, S2). The UPB and LPB energies are extracted from reflectivity measurements as a function of position on the cavity and fit at each detuning with Eq. 1^1^, yielding an excellent fit across detunings to the theoretical UPB and LPB energies (Fig. 1e, ${{{\hslash }}\Omega }_{{Rabi}}$ = 260 meV, SI).

1 $${E}_{{{{{{\rm{LP}}}}}},{{{{{\rm{UP}}}}}}}\left({k}_{{{{{{\rm{||}}}}}}}\,\right)=\frac{1}{2}\left[{E}_{{{{{{\rm{exc}}}}}}}+{E}_{{{{{{\rm{cav}}}}}}}({k}_{{{{{{\rm{||}}}}}}})\pm \sqrt{{4{g}_{0}^{2}+\left({E}_{{{{{{\rm{exc}}}}}}}-{E}_{{{{{{\rm{cav}}}}}}}({k}_{{{{{{\rm{||}}}}}}})\right)}^{2}}\,\right]$$

where ${E}_{{LP},{UP}}\left({k}_{{||}}\right)$ corresponds to the upper and lower polariton branch energies as a function of *k*_*||*_, ${E}_{{exc}}$ is the exciton energy (considered dispersionless over *k*_*||*_ values measured), ${E}_{{cav}}({k}_{{||}})$ is the uncoupled cavity energy as a function of *k*_*||*_, and $2{g}_{0}={{{\hslash }}\Omega }_{{Rabi}}$ is the normal mode splitting, or Rabi splitting as in a single-atom microcavity system^1^.

The Hopfield coefficients for each dispersion quantify the excitonic and photonic fraction as a function of *k*_*||*_ (Eq. 2), with the most excitonic detuning possessing >50% excitonic character at *k*_*||*_ = 0 and the most photonic detuning possessing >50% photonic character at *k*_*||*_ = 0 (Figs. 1a–c, S3, S4 lower panels).

2 $${\left|{X}_{k}\right|}^{2},{\left|{C}_{k}\right|}^{2}=\,\frac{1}{2}\left(1\pm \frac{\triangle E({k}_{{{{{{\rm{||}}}}}}})}{\sqrt{{\triangle E({k}_{{{{{{\rm{||}}}}}}})}^{2}+4{g}_{0}^{2}}}\right)$$

where ${\left|{X}_{k}\right|}^{2}$ and ${\left|{C}_{k}\right|}^{2}$ are the excitonic and photonic Hopfield coefficients, respectively.

As the cavity mode shifts to lower energy and the polariton dispersion becomes more photonically detuned, the emission distribution shifts from the maximum PL intensity at *k*_*||*_ = 0 to higher *k*_*||*_ (Fig. 1f, g), known as the polariton bottleneck regime, a well-studied effect in inorganic and organic polariton systems^29,30^.

The bottleneck effect is a manifestation of the reduced scattering rate of high *k*_*||*_ polaritons as they relax down the LPB. This effect is a combined consequence of the decreased excitonic character as polaritons move down their dispersion curve, which makes exciton-phonon scattering less efficient, and of the reduced density of states as the polariton effective mass decreases, that is, as the LPB curvature increases. Additionally, an increase in photonic character is accompanied by an increase in radiative rates, which leads to the depletion of the polariton population near *k*_*||*_ = 0 if the scattering rates into nearby states is too low^29^. As the overall excitonic character of polaritons diminishes, and as the LPB curvature increases, the bottleneck effect becomes more pronounced for negative (photonic) detunings. The polariton lifetime at negative detunings is longer than at positive detunings due to the increase in excitonic character of polaritons above the bottleneck region, consistent with our measurements in this system (Fig. S5).

The polariton bottleneck effect has been shown to be thermally activated—by decreasing the system temperature, exciton-phonon scattering is similarly reduced, resulting in the emergence of the bottleneck even for polaritons with mostly excitonic character^30^. This phenomenon is difficult to study in systems that require cyrogenic temperatures to achieve strong coupling (e.g., GaAs heterostructures), but room-temperature strong coupling systems present an opportunity to investigate the role of phonon scattering in polariton relaxation by lowering the temperature. Thus, we are able to explore the impact of LO phonon-exciton Fröhlich interactions and acoustic phonon-induced deformation potential mechanisms that can accelerate or hamper key polariton scattering pathways^31^.

### Temperature-dependent polariton photophysics in perovskite microcavities

By reducing the microcavity temperature and carefully controlling the cavity length along the cavity wedge to modulate the detuning, we explore the emergence of the bottleneck regime for polaritons with varying excitonic/photonic character and thus changing energy differences between the exciton reservoir and the bottom of the lower polariton branch. Additionally, the impact of the magnitude of photon-exciton coupling on temperature-dependent scattering mechanisms is investigated by modifying the Rabi splitting to determine the contribution of enhanced coupling strength on polariton relaxation toward *k*_*||*_ = 0.

**Fig. 2:**
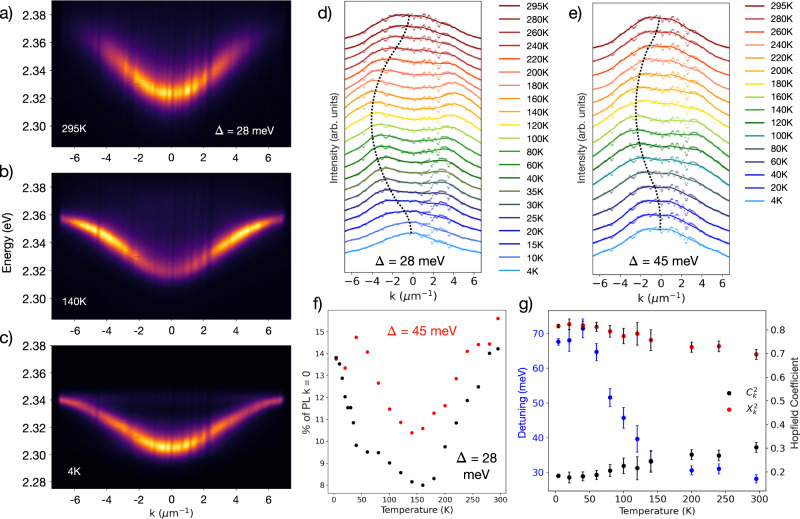
Temperature dependence of the exciton-polariton bottleneck effect. **a**–**c** Lower polariton branch (LPB) photoluminescence (PL) for ∆ = +28 meV cavity detuning as a function of temperature revealing **d**, **e** the shifting of the maximum PL intensity to higher *k*_*||*_ at intermediate temperatures before returning to *k*_*||*_ = 0 at 4 K. **d**–**f** The temperature-dependence of the energy-integrated PL was monitored for two detunings established at room temperature (${{{\hslash }}\Omega }_{{Rabi}}$ = 175 meV, ∆ = +28 meV and +45 meV), with both detunings showing bottlenecked PL at intermediate temperatures and emission from *k*_*||*_ = 0 at sufficiently low temperatures (raw data, dashed trace; smoothed data, solid trace; peak PL trend to guide the eye, symmetric about *k*_*||*_ = 0, dotted trace). **g** Cavity detuning and Hopfield coefficients as a function of temperature for ∆ = +28 meV (fit error as determined by RSS), revealing that the detuning becomes more positive as temperature decreases with polaritons shifting from 70% excitonic at 295 K to 80% excitonic at 4 K.

To begin deconvoluting these various relaxation mechanisms, we explore the temperature-dependent *k*-space PL distribution for two coupling strengths: ${{{\hslash }}\Omega }_{{Rabi}}$ = 175 meV (Fig. 2) and ${{{\hslash }}\Omega }_{{Rabi}}$ = 260 meV (Fig. S6) by changing the perovskite active layer thickness (i.e., number of oscillators $N$, ${{{\hslash }}\Omega }_{{Rabi}}\propto \,\sqrt{N}$)^32^. Additionally, temperature cycles are performed on two detunings (∆ = +28 meV and +45 meV). For both positive cavity detunings with emission from *k*_*||*_ = 0 at 295 K, we probe the emergence of the bottleneck region with decreasing temperature due to the reduction of phonon scattering pathways.

We begin to observe the redistribution of PL to high *k*_*||*_ at ~200 K, with the most pronounced bottleneck at ~140 K (Fig. 2). Unexpectedly, the PL distribution in k-space for ∆ = +28 meV re-centers at *k*_*||*_ = 0 for temperatures below 15 K, and, for ∆ = +45 meV, re-centers to *k*_*||*_ = 0 for temperatures below 60 K (Fig. 2d, e). For both detunings, we see the appearance and suppression of the bottleneck effect, with PL moving away from *k*_*||*_ = 0 when cooling down to 140 K and shifting back to *k*_*||*_ = 0 when cooling down to 4 K (Figs. 2f, S6f). To understand this behavior, we note several key factors at play with decreasing temperature, both for the perovskite film system in isolation and for the strongly coupled microcavity system.

If there are temperature-dependent changes in the bare excitonic thin film outside of the microcavity (e.g., structural changes, bandgap and concomitant emission energy shifts, PLQE changes), these effects could manifest as temperature-dependent alterations in the polariton density and therefore polariton-polariton scattering rates. For the bare 2D film, we confirm that there are no significant changes in the perovskite structure (i.e., phase change) as a function of temperature (Fig. S7), and, as previously shown in PEA_2_PbI_4_ 2D perovskites, we observe a redshift in the PL with decreasing temperature which is consistent with the Varshni effect^33^. Additionally, we quantify a ~100-fold increase in PLQE, from ~0.7% at 295 K (consistent with other reports)^34^ to between 30% and 40% as the temperature drops from 180 K to 40 K to 77% at 4 K (Fig. S8). While the increase in film PLQE as the temperature decreases serves to increase the polariton population and thus should enhance polariton-polariton scattering, this effect is likely not the dominant relaxation mechanism for *k*_*||*_ = 0 emission at 4 K, as we see very little change to the *k*-space PL distribution as a function of excitation power spanning five orders of magnitude (Figs. S9, S10). If enhanced polariton–polariton scattering due to the increase in material PLQE were the dominant mechanism for relaxation to *k*_*||*_ = 0 at low temperature, we would expect the re-emergence of the bottleneck effect at sufficiently low excitation powers, or low polariton densities, which we do not observe. Even at low fluences (~30 nJ/cm^2^/pulse), efficient relaxation to *k*_*||*_ = 0 at 4 K is achieved.

For the microcavity system, the exciton PL redshift (~5 nm), combined with the thermal contraction of the microcavity due to cavity cooling (primarily due to the shrinking of the optically inert organic spacer layer) results in an overall blue-shift of the cavity energy (~3 nm, Fig. S11), which leads to progressive increases in the detuning with decreasing temperature, from ∆ = +28 meV at 295 K to ∆ = +68 meV at 4 K, or from 70% excitonic character to 80% excitonic character (Fig. 2g). This change in detuning alone is not responsible for the re-centering of the PL distribution around *k*_*||*_ = 0; we are uniquely able to hold the detuning constant by leveraging the radial wedged cavity and moving to lower energy cavity modes at lower temperatures, thus showing that the bottleneck effect and its suppression are observed even when the cavity detuning is fixed (Fig. S12).

With the above mechanisms likely not dominant contributors to the emergence and supression of the bottleneck effect, we look to better understand how various excitonic species may play a role in polariton formation and relaxation by probing the emergence of biexciton and dark exciton emission as a function of decreasing temperature.

### Bright and dark excitons in bare 2D perovskite thin films

**Fig. 3:**
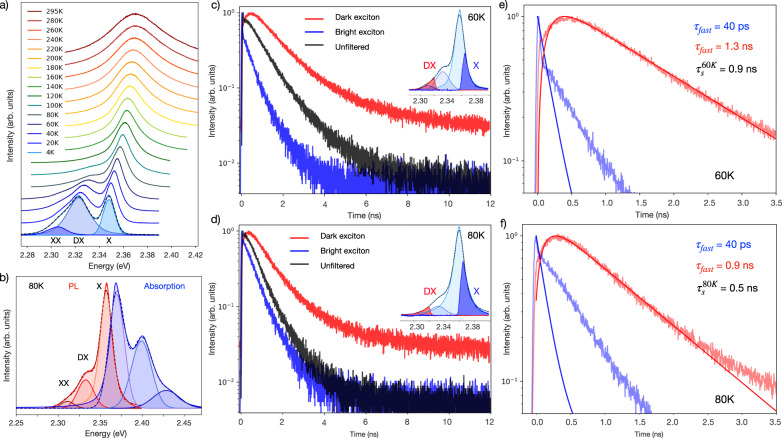
Temperature-dependent behavior of excitonic species in the thin film. **a** 2D perovskite thin film photoluminescence (PL) spectra as a function of temperature revealing, beyond the primary PL peak, the emergence of secondary and tertiary PL peaks assigned to **b** the bright exciton (X), the dark exciton (DX), and the biexciton (XX) with PL shown in red and absorption in blue. **c**, **d** Time-resolved PL for the unfiltered bare 2D film spectrum (black), X filtered spectrum (blue), and DX filtered spectrum (red). Insets: 2D perovskite thin film PL at **c** 60 K and **d** 80 K with fitted Gaussian constituent peaks. High (dark blue) and low (dark red) energy regions highlighted, showing the experimentally measured PL spectra with tunable edge-pass filters for X and DX decay measurements, respectively. **e**, **f** Lifetimes of the X emission (blue) and DX emission (red) simulated with Eqs. 3–5.

The luminescence spectrum of the uncoupled, bare 2D perovskite film at room temperature (295 K) shows a dominant bright exciton (X) peak at 2.370 eV (Fig. 3a). As the film is cooled, the PL monotonically red-shifts, reaching a center energy of 2.348 eV at 4 K (Fig. S13).

A second, low-energy peak emerges below ~140 K, which is consistent with reports of a dark exciton (DX) with center energy 2.323 eV at 4 K (Figs. 3a, S13), and has been shown to increase in PL intensity in the presence of an external magnetic field^35–37^. Emission of the DX state in the absence of the magnetic field has also been observed due to spin-orbit coupling and dipole mixing with the bright exciton (X)^36,37^. Though the films posses a high degree of crystallinity (Fig. S7), we are unable to resolve the four states previously reported within the fine structure for the bright and dark excitons due to PL broadening resulting from disorder of the polycrystalline thin film^35–38^.

Below 80 K, we observe a third, low energy peak below the DX (center energy 2.305 eV at 4 K, Fig. S13), which can be attributed to either electron-phonon coupling, self-trapped excitons, and/or to biexcitons (XX) (Fig. 3a)^35–39^. Given earlier reports, we attribute this third, low energy peak in the PL to the biexciton, for which we calculate a biexciton binding energy of $\Delta {E}_{{XX}}=\,$4  meV, utilizing PL as a proxy for state energies and relative energies, in agreement with Thouin et al.^40^. The increase in biexciton emission with decreasing temperature is expected with the observed increase in the radiative luminescence efficiency, and hence the increase in the radiative exciton density.

Though multiple excitonic species are visible in the low-temperature 2D film PL, single-mode polariton dispersions are observed down to 4 K, direct evidence that the cavity mode strongly couples to only one exciton species^41^. To better understand the polariton photophysics and excitonic state to which the cavity couples at low-temperature, we investigate the bare 2D film bright, X, and dark, DX, state dynamics by selecting high and low energy regions of the film PL spectrum with spectral filters, capturing separately the decay of X and DX, respectively (Fig. 3). Furthermore, to separate the DX and XX response, as they are in the same low energy region, these measurements are performed at 80 K and 60 K, temperatures at which DX emission is present and the XX emission is not yet significant.

We observe delayed emission from DX (low energy spectral region, Fig. 3e, f), which is strong evidence for a spin-flip process^42,43^ facilitated by the dark exciton gaining oscillator strength from the bright exciton by mixing due to spin-orbit coupling^37,44^, and by limiting the non-radiative phonon-assisted relaxation at low temperatures. This transfer process can be modeled by a set of coupled differential equations. These equations capture the three-state model, in which radiative recombination from the bright exciton (denoted as *X* in Eqs. 3–5) competes with transfer to the dark exciton population (denoted as *DX* in Eqs. 3–5) and vice versa through microscopic reversibility^45^. The temperature-dependent spin-flip rate (${k}_{s}(T)$) and the reverse process (${k}_{s-}(T)$) maintain thermodynamic equilibrium, weighted by the Arrhenius factor: ${k}_{s-}\,{={e}^{({E}_{X}-{E}_{{DX}})/{kT}}\cdot k}_{s}$ (Fig. S14b)^45,46^.

To fully capture the exciton/photon dynamics in the photoexcited 2D perovskite film, we take into account photon recycling due to the low material Stokes shift and high absorption (Figs. 3b, S15, S16), creating additional terms that contribute to the excited state dynamics through radiative recombination of either a bright or dark exciton (Eqs. 3, 5)^47,48^.

3 $$\frac{d{n}_{X}}{{dt}}=-{k}_{{rX}}{n}_{X}-{k}_{s}{\left(T\right)n}_{X}+{k}_{s-}(T){n}_{{DX}}+\frac{c}{{n}_{r}}\mathop{\sum}\limits_{\lambda }{\alpha }_{\lambda }{\gamma }_{\lambda }$$

4 $$\frac{d{n}_{{DX}}}{{dt}}=-{k}_{{rDX}}{n}_{{DX}}+{k}_{s}{(T)n}_{{DX}}-{k}_{s-}{(T)n}_{{DX}}$$

5 $$\frac{d{\gamma }_{\lambda }}{{dt}}=-\frac{c}{{n}_{r}}\mathop{\sum}\limits_{\lambda }{\alpha }_{\lambda }{\gamma }_{\lambda }+\left(1-{P}_{{{{{{\rm{esc}}}}}}}\right)\cdot \left[{k}_{{rX}}{n}_{X}+{k}_{{rDX}}{n}_{{DX}}\right]$$

where ${k}_{{rX}}$ and ${k}_{{rDX}}$ are the radiative recombination of X and DX, respectively, ${k}_{s}(T)$, and ${k}_{s-}(T)$ are the temperature-dependent $(T)$ spin-flip rates from X to DX and DX to X allowing for interconversion between both species, respectively, ${n}_{X}$ and ${n}_{{DX}}$ are the X and DX energy carrier concentrations, respectively, $c$ is the speed of light, ${\alpha }_{\lambda }$ is the absorption coefficient averaged over the emission band, ${\gamma }_{\lambda }$ is the photon concentration within the film for a given wavelength due to radiative recombination and photon recycling, ${n}_{r}$ is the index of refraction, and ${P}_{{{{{{\rm{esc}}}}}}}$ the probability of a radiatively recombined photon leaving the film within the escape cone.

In this way, ${k}_{s}(T)$ can be quantified as a function of temperature in this material system. At 60 K (Fig. 3c, e), the system of coupled differential equations yields a fast bright exciton lifetime ${\tau }_{60{{{{{\rm{K}}}}}},{{{{{\rm{fast}}}}}}}^{X}$ < 40 ps, limited by the instrument response function (IRF) of the detection scheme (Fig. S17). The long tail of the time-resolved photoluminescence (TRPL) trace is not included in the model given system IRF limitations and the non-zero spectral overlap of the dark exciton contributing to the counts at longer timescales. The dark exciton has a fast component, ${\tau }_{60{{{{{\rm{K}}}}}},{{{{{\rm{fast}}}}}}}^{{DX}}$ = 1.3 ± 0.05 ns, and long tail, fit with an exponential decay, of ${\tau }_{60{{{{{\rm{K}}}}}},{{{{{\rm{slow}}}}}}}^{{DX}}$ = 15 ± 3 ns (Fig. S18), which is consistent with reports of the long DX lifetime in these materials^37^. The transfer process at 60 K, ${\tau }_{60{{{{{\rm{K}}}}}}}^{s}$ = 0.9 ± 0.15 ns, is relatively slow^49^, but several studies show similarly long time-scales for spin dynamics in perovskites and other materials at low temperature^50–52^.

When the temperature is increased from 60 K to 80 K, the lifetime of the bright exciton remains IRF-limited (${\tau }_{80{{{{{\rm{K}}}}}},{{{{{\rm{fast}}}}}}}^{X}$ < 40 ps), the dark exciton fast-component lifetime decreases (${\tau }_{80{{{{{\rm{K}}}}}},{{{{{\rm{fast}}}}}}}^{{DX}}$ = 0.9 ± 0.05 ns), and the transfer rate increases (${\tau }_{80{{{{{\rm{K}}}}}}}^{s}$ = 0.5 ± 0.15 ns), which is anticipated considering the Arrhenius relation and large bright-dark state splitting in this material, and is consistent with a thermally activated spin-flip (Fig. 3d, f)^45,46^.

Both X and DX states can be populated following photoexcitation, with additional DX population contributions due to transfer from the higher energy X to DX at rate ${k}_{s}(T)$, and additional X population due to the thermally dependent back-transfer from DX to X at rate ${k}_{s-}(T)$^45,53^. As temperature decreases and non-radiative phonon scattering events are reduced, luminescence of the long-lived DX population can outcompete the phonon-assisted non-radiative emission resulting in the observable DX emission peak. Additionally, the bi-directional interconversion between X and DX (${k}_{s}(T)$, ${k}_{s-}(T)$), as previously demonstrated in many material systems^45^, favors the population of the lower-lying DX as temperature decreases. We calculate$\,{\tau }^{s-}=\,1/{k}_{s-}(T)$ to be ${\tau }_{80{{{{{\rm{K}}}}}}}^{s-}$ = 63 ns and ${\tau }_{60{{{{{\rm{K}}}}}}}^{s-}$ = 110 ns, which is much slower than the measured radiative emission time of the DX state.

Thus, we observe a steady increase in the DX emission with decreasing temperature due to both a reduction in non-radiative phonon scattering and the Arrhenius decrease of thermal excitation from DX to X, as seen in similar systems^54^. Additionally, the impact of photon recycling on these dynamics is non-trivial, as for a PLQE of 77% at 4 K, the average number of recycling events per emitted photon in the 2D perovskite thin film is 3.5, with recycling events increasing to 13 in the radiative limit (Fig. S15). Photon recycling effectively functions as a mechanism for repopulating the bright state exciton reservoir, allowing another opportunity for the regenerated bright excitons to spin-flip into the dark state.

### Luminescence lifetimes of excitons and cavity LPB states

**Fig. 4:**
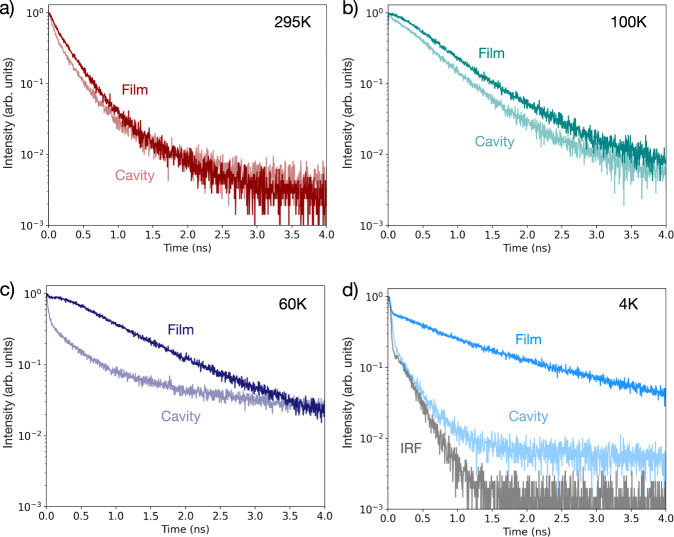
Temperature-dependent thin film and strongly coupled microcavity dynamics. **a**–**d** The bare 2D perovskite film time-resolved photoluminescence (TRPL) decay (dark traces, Film) compared to the strongly coupled microcavity (${{{\hslash }}\Omega }_{{Rabi}}=175\,{{{{{\rm{meV}}}}}}$, ∆ = +28 meV) lower polariton branch (LPB) emission (light traces, Cavity) as a function of temperature (295 K, 100 K, 60 K, 4 K). In the bare film, as temperature decreases, the bright exciton (X) emission lifetime decreases and the dark exciton (DX) emission emerges with an increasingly long lifetime, visible as a short-timescale fast component with delayed emission into a longer tail ((**b**, **c**), dark traces). In the cavity (**b**–**d**, light traces), the extent of delayed emission is reduced. At 60 K in the cavity (**c**, light trace), the fast LPB emission begins to dominate the TRPL decay dynamics at early timescales, with weaker emission contribution from the DX state resulting in a long lifetime tail. (**d**, light trace) At 4 K in the cavity, a nearly IRF-limited decay is observed with no long lifetime contribution.

Figure 4 shows the temperature-dependent emission lifetimes for the bare 2D film together with the lifetimes of the strongly coupled cavity (additional temperature-dependent TRPL traces are shown in Fig. S19-S20). At room temperature, the bare 2D film luminescence lifetime is ${\tau }_{295{{{{{\rm{K}}}}}}}=350$ ± 20 ps, and the LPB lifetime is ${\tau }_{295{{{{{\rm{K}}}}}},{{{{{\rm{cav}}}}}}}=260$ ± 20 ps, representative of the transient response near the cavity polaritons (Fig. 4a). This decay is longer than the expected polariton lifetime (<100 fs for Q ~ 110) due to the influence of reservoir states that are not strongly coupled and only weakly perturbed by cavity polaritons^13^ as well as by photoinduced effects unique to the cavity system^55^. For example, in organic thin films, it has been shown that the polariton lifetime of low-Q cavities, intrinsically on the order of 10’s of fs, in practice follows the time-evolution of the fundamental carrier and spin dynamics of material excitons on much longer timescales (ns to µs in duration), resulting in strongly coupled PL decay dynamics that are similar to the bare film exciton dynamics^13^.

In the bare 2D film, as the temperature decreases from 295 K to 100 K, the X emission lifetime decreases and the DX emission emerges with an increasingly long lifetime and increasingly strong intensity, as has previously been observed by Fang et al. (Fig. 4b, dark teal trace, 100 K)^38^. Further reductions in temperature show that, as the back transfer ${k}_{s-}(T)$ slows, the X emissive lifetime shortens and its early timescale emission increases, while DX demonstrates an increasingly long emissive lifetime (Fig. 4c, d, dark purple and blue traces). Indeed, by 4 K, the DX state constitutes most of the emission of the bare perovskite film, with a 100-fold increase in the overall luminescence efficiency, as compared to the PLQE at 295 K.

The improvements in the perovskite film PLQE and the emergence of significant emission from the DX state (Fig. 3) occur over the same temperature range in which the cavity LPB dispersion undergoes its own spectral emission changes (Fig. 2). In the strongly coupled cavity at 100 K, the extent of delayed emission is similar to but slightly reduced from the bare 2D film, and the fast component contribution increases as the strong coupling between the bright exciton and cavity mode forms the short-lifetime LPB emissive state (Fig. 4b, light teal trace, 100 K). As the temperature decreases from 60 K to 4 K in the cavity, the fast emission from the LPB shifts in energy (Fig. 2) and the emission rate increases (Fig. 4c, light purple trace). At 4 K in the cavity, we measure nearly IRF-limited emission largely from the strongly coupled state emitting near *k*_*||*_ = 0 and do not observe significant delayed emission or the long tail from the DX emission (Fig. 4d, light blue trace).

### Dark exciton intracavity pumping and biexciton-assisted relaxation

While the strong coupling between the bright exciton, X, and cavity mode results in a short polariton emissive lifetime, there can still exist the DX population inside the cavity, generated after photoexcitation but not strongly coupled to the cavity, consistent with the weak oscillator strength and dipole misorientation of the DX state^56,57^. The brightening of the DX state at lower temperatures allows its luminescence to then be observed via the LPB mode, which is resonant in energy with the DX state. Decay of a lower energy DX state through the resonant LPB mode has been observed in strongly coupled organic microcavities^58^, and increased polariton density has been demonstrated by cavity-enhanced, *k*_*||*_-dependent transfer of the DX population to the LPB^59^ as well as the radiative pumping of polariton modes by a secondary, uncoupled or weakly coupled isoenergetic emitter within the cavity^15,60–62^.

**Fig. 5:**
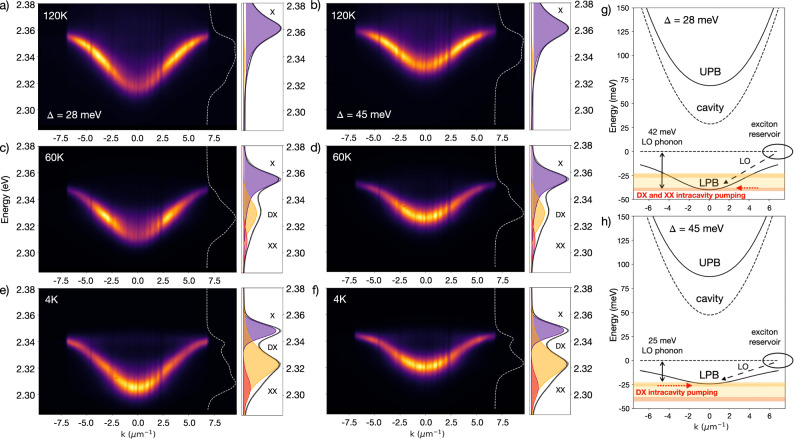
Exciton-polariton relaxation schemes towards bottleneck suppression. **a**–**f** Lower polariton branch (LPB) photoluminescence (PL) as a function of temperature for two detunings established at room temperature (∆ = +28 meV and +45 meV) with (right panel) bare 2D perovskite film PL at the corresponding temperature (bright exciton = X; dark exciton = DX; biexciton = XX). **a**, **c**, **e** For the more photonic detuning (∆ = +28 meV), the re-centering of the PL distribution to *k*_*||*_ = 0 occurs when the bottom of the LPB is resonant with XX due to **g** intracavity pumping and the 42 meV LO-phonon-mediated relaxation. **b**, **d**, **f** For the more excitonic detuning (∆ = +45 meV), the re-centering of the PL distribution to *k*_*||*_ = 0 occurs when the bottom of the LPB is resonant with DX due to **h** intracavity pumping and the 25 meV LO-phonon-mediated relaxation.

In our system, as the temperature of the cavity drops from 100 K to 4 K, the higher energy *k*_*||*_ > 0 LPB population can resonantly transfer to the luminescent DX state due to weak coupling facilitated by the DX acquiring oscillator strength at high-*k*_*||*_^59^. The energy in the DX state can be rapidly transferred back to the isoenergetic LPB state (Fig. 5), creating a new pathway of emission as compared to the long-lived DX state in the bare 2D film case. The DX state can then also be described as an intermediary that shuttles the *k*_*||*_ > 0 LPB energy to the *k*_*||*_ state of the same energy as the DX. When the bottom of the LPB, or *k*_*||*_ = 0 mode, is resonant with the DX state, the result is the suppression of the bottleneck effect with primary emission from *k*_*||*_ = 0 (Fig. 5d,f). A similar isoenergetic release of, or radiative pumping by, the XX luminescence through the LPB mode can also be sustained at sufficiently low temperatures when the XX state emits (Fig. 5e). Radiative pumping of the LPB by the biexciton has been previously observed by Polimeno et al. in PEA_2_PbI_4_ microcavities at sufficiently high fluence, representing a threshold of emission redistribution reached before condensation at higher pump fluences (See SI for additional discussion)^63^.

Additionally, while the cavity does not strongly couple to the DX or XX states, transitions between these species and bright excitons appear resonant with LO-phonon modes in PEA_2_PbI_4_ as computed by Straus et al. for energies below 50 meV (400 cm^−1^) using DFT calculations^39^. Straus et al. quantify mode contributions from the high-energy organic cation vibrations (25 meV and 41 meV) that are resonant with the DX and XX energy differences from the bright exciton (X-DX = 25 meV, X-XX = 42 meV)^39^. In this work, we excite above band gap, and generate hot carriers that can cool via nonradiative relaxation with vibronic replicas—states that resonantly emit phonons into the XX state and DX state during spin-flip transfer processes^64,65^. These phonon modes are always present, but it is only when the red-shifting exciton reservoir and corresponding LPB minimum is resonant with a transition involving one of these phonon modes at low temperature that LO-phonon assisted relaxation can enhance scattering processes (Fig. 5). When the detuning is such that the energy difference between the bottom of the LPB and the exciton reservoir matches the energy of one LO-phonon, efficient LO-phonon-mediated relaxation pathways may be utilized.

Emission from *k*_*||*_ = 0 at low temperature can then be described as a combined consequence of intracavity pumping from the DX and XX populations, resonant LO-phonon scattering, and biexciton-assisted relaxation. For ∆ = +28 meV at 120 K (Fig. 5a), the brightest emission is observed at high *k*_*||*_ (bottleneck), whereas, at 60 K, the brightest emission has shifted to lower *k*_*||*_, peaked at the resonant DX energy and benefitting from isoenergetic intracavity pumping with further opportunity for bottleneck suppression via X-DX LO-phonon scattering (Fig. 5c). At 4 K, the LPB minimum red-shifts, becoming resonant with the XX state, and the LPB emission profile redistributes due to the isoenergy of the XX state and *k*_*||*_ = 0 mode, with available scattering pathways from relaxation via X-XX LO-phonons (Fig. 5e, g).

For ∆ = +45 meV at 120 K (Fig. 5b), the bottleneck effect is again observed, though to a lesser extent as the increased excitonic character reduces the curvature of the LPB and increases the DOS, allowing for greater scattering towards *k*_*||*_ = 0. Because of the flatter dispersion, the bottom of the LPB never reaches the XX energy resonance, but instead is resonant with the DX state and again matches the smaller LO-phonon energy difference from X (X-DX). This occurs at higher temperatures than its less excitonic counterpart, ∆ = +28 meV, resulting in *k*_*||*_ = 0 emission at an elevated temperature of 60 K (Fig. 5d), maintaining *k*_*||*_ = 0 primary emission down to 4 K (Figs. 5f, h, S21–25).

In this work, we have identified the interplay between PEA_2_PbI_4_ perovskite excitonic states and exciton-polariton formation as a function of temperature in wedged microcavity systems. By careful control of the Hopfield coefficients, the impact of changing cavity detuning and thus changing polariton photonic/excitonic character on the lower polariton branch (LPB) emission profile in *k*-space was determined. We have shown that the bottleneck effect emerges at intermediate temperatures and can then be suppressed at low temperatures with the emergence of dark exciton (DX) and biexciton (XX) luminescence. Isoenergetic DX and XX intracavity pumping can be assisted by LO-phonon-mediated scattering and biexciton-assisted polariton relaxation for *k*_*||* _= 0 emission in this 2D perovskite exciton-polariton system, presenting opportunities for engineering the microcavity detuning such that the energy minimum of the lower polariton branch can be directly populated. By targeted synthetic, passivation, or applied external field approaches, the brightening of these excitonic states can be harnessed to increase the polariton population at elevated temperatures^66–69^. In this way, we demonstrate, without synthetic modifications, tuning of the perovskite electronic structure via strong coupling to enable new kinetic rates. These insights provide cavity and material design principles for next-generation polaritonic devices requiring careful control of polariton momentum and relaxation, and demonstrate the utility of polariton formation to non-synthetically modify recombination dynamics and energy conversion processes towards optoelectronic devices with tunable emissive properties.

## Methods

### Perovskite preparation

Perovskite precursors were obtained from Sigma Aldrich (phenethylammonium iodide, SKU 805904) and TCI (lead(II) iodide, TCI-L0279), and prepared in dimethyl sulfoxide (Sigma Adlrich, SKU 34869) stoichiometrically for *n* = 1. Films were spincast in a two-step procedure: (1) 1000 rpm, 10 s, 500 accel; (2) 5000 rpm, 30 s, 2000 accel with a chlorobenzene quench 15 s before the end of the second step (Sigma Aldrich, SKU 284513). The films were annealed at 100 °C for 10 min. All synthesis and process steps under nitrogen. The resulting thin films ranged from approximately 25–50 nm (± approximately 2 nm, ellipsometry) as a function of solution concentration, as measured on silicon substrates by ellipsometry.

### Solution-processed spacer layer preparation

Poly(methyl methacrylate) was purchased from Sigma Aldrich (SKU) and dissolved in chlorobenzene (Sigma Aldrich, SKU 284513) at 50 °C. Films were spincast using a single-step procedure: 1500 rpms, 60 s, 1000 accel. Films were gently annealed at 60 °C for 1 min to assist in driving off excess solvent. All process steps under nitrogen. The resulting thin films were approximately 110 nm thick, as measured on fused silica substrates by profilometry (RMS = 1 nm, profilometry, radial variation measured optically within the microcavity to be ~30 meV/mm).

### Microcavity preparation

Fused silica substrates were cleaned by sonication in water, diluted detergent, acetone, and isopropyl alcohol followed by boiling isopropyl alcohol. The bottom Ag mirror was thermally evaporated at 110 nm followed by sputter deposition of a 108 nm SiOx layer in argon (RMS of Ag and SiOx layer = 2 nm, profilometry). The perovskite active layer was spin-cast and annealed under nitrogen (~25 nm), followed by the Poly(methyl methacrylate) layer. The microcavity was capped with a semi-transparent thermally evaporated Ag layer (35 nm).

### Room-temperature Fourier spectroscopy

K-space emission was imaged using a Nikon Eclipse-Ti inverted microscope fitted with an infinity corrected $100\times$ dry objective (Nikon L Plan, NA = 0.85). A 405 nm pulsed diode laser (PDL-800 LDH-P-C-405B, 300 ps pulse width) was used for excitation with repetition rate of 80 MHz. The sample photoluminescence (PL) was filtered through a 405 nm dichroic beamsplitter (Nikon DiO1-R405) and the reflectivity collected via a halogen lamp white light source (Nikon Eclipse-Ti) and 50/50 beamsplitter (Chroma 21014-UF3 C188781). The output for both PL and reflectivity was then coupled in free space via a 4 F imaging system into a Princeton Instruments Acton spectrometer and Pixis camera (100 (k-space) × 1340 (wavelength) pixels).

### Low-temperature (4-295 K) Fourier spectroscopy

K-space emission was imaged using a Montana Instruments closed-cycle liquid He crysotat with piezo-controlled 3D-moveable sample stage, cryo-optic low-working distance 100x 0.9NA objective, vacuum housing, radiation shield, and local objective heater. A wavelength-tunable ultrafast laser (Toptica Photonics FemtoFiber Pro) was used for 488 nm excitation with 80 MHz repetition rate, guided into the cryo-optic with electrically-controlled Thorlabs Galvo mirrors. The sample emission was filtered through a Semrock tunable edge pass (set to 490 nm long pass) filter and directed via a 4 F imaging system into a Princeton Instruments Acton spectrometer and either a 512 (k-space) × 512 (wavelength) pixel or 1024 (k-space) × 1024 (wavelength) pixel Pixis camera.

### Time-resolved photoluminescence

A wavelength-tunable ultrafast laser (Toptica Photonics FemtoFiber Pro) was used for 488 nm excitation with 80 MHz repetition rate. The emission was collimated and filtered through a Semrock tunable edge pass (set to 490 nm long pass) filter and focused onto a Micro Photon Devices (MPD) PicoQuant PDM Series single photon avalanche photodiode with a 50 μm active area and 40 ps IRF. Photon arrival times were time-tagged using a time-correlated single photon counter (TimeHarp 260).

### Variable angle spectroscopic ellipsometry

Spectroscopic ellipsometry was performed using a variable angle spectroscopic ellipsometer (Woollam) at 65°, 70°, and 75° angles of incidence. Ellipsometry data was fitted to obtain perovskite thin film thicknesses.

### Photoluminescence quantum efficiency (PLQE) measurements

PLQE measurements were acquired using a center-mount integrating sphere setup (Labsphere CSTM-QEIS-060-SF) and Ocean Optics USB-4000 spectrometer. The integrating sphere setup was intensity calibrated with a quartz tungsten halogen lamp (Newport 63355) with known spectral irradiance set at a distance 0.5 m away from the integrating sphere illumination port. A fiber-coupled 405 nm diode laser in CW mode (PDL-800 LDH-P-C-405B) was collimated with a triplet collimator (Thorlabs TC18FC-405) to produce a beam with an approximate 1/e^2^ diameter of 2.8 mm. The beam was used to excite the sample and a variable neutral density filter was used to attenuate the laser. Data acquisition followed the protocol described by de Mello et al.^70^, with a scattering correction.

### Cryo XRD

Temperature-dependent XRD was performed using a Panalytical Multipurpose Diffractometer with a liquid He cryostat for in-situ low temperature measurements. Around 30 min scan was taken for 5–67° at each temperature in increments of 20 K from 295 K to 11 K, with 15 min between scans for temperature equilibration. The temperature was scanned from high to low and cycled back from low to high to determine whether the temperature cycle damaged the 2D perovskite. No structural changes (peak intensity or position) were noted in the upcycle.

### Cryo absorption

Reflection spectrophotometry was performed with light incident from the film side using an Agilent Cary 5000 dual-beam UV–vis–NIR spectrophotometer with home-built, liquid N_2_ cryostat quartz window attachment. The 2D perovskite was coated onto a 15 mm diameter fused silica optical window (see substrate cleaning procedure above) for compatibility with cryo sample holder. Specular reflectance was collected at an incident angle of 8°. A 3 mm round aperture was used for all measurements.

## Supplementary information

Supplementary Information

## Data Availability

The data that support the findings of this study are available from the first author and corresponding authors on request. Source data are provided with this paper.
