# Supplementary material for: Uncovering temperature-dependent exciton-polariton relaxation mechanisms in hybrid organic-inorganic perovskites
Source: Nat Commun. 2023 Apr 27;14:2426. doi: 10.1038/s41467-023-37772-7 (PMC10140020; doi:10.1038/s41467-023-37772-7)
Supplement: Supplementary file 1 — Supplementary Information [file 41467_2023_37772_MOESM1_ESM.pdf]

Supporting Information

Uncovering Temperature-Dependent Exciton-Polariton Relaxation Mechanisms in Hybrid Organic-Inorganic Perovskites

Madeleine Laitz[1], Alexander E. K. Kaplan[2], Jude Deschamps[2], Ulugbek Barotov[2], Andrew H. Proppe[2], Inés García-Benito[3], Anna Osherov[1], Giulia Grancini[4], Dane W. deQuilettes[5*], Keith A. Nelson[2], Moungi Bawendi[2], Vladimir Bulović[1*]

[1]Department of Electrical Engineering and Computer Science, Massachusetts Institute of Technology, 77 Massachusetts Avenue, Cambridge, Massachusetts 02139, USA

[2]Department of Chemistry, Massachusetts Institute of Technology, 77 Massachusetts Avenue, Cambridge, Massachusetts 02139, USA

[3]Department of Organic Chemistry, Universidad Complutense de Madrid. Av. Complutense s/n. 28040 Madrid, Spain.

[4]Department of Chemistry & INSTM, University of Pavia, Via Taramelli 14, 27100 Pavia, Italy

[5]Research Laboratory of Electronics, Massachusetts Institute of Technology, 77 Massachusetts Avenue, Cambridge, Massachusetts 02139, USA

*Corresponding Authors: danedeq@mit.edu, bulovic@mit.edu

Strongly coupled perovskite microcavities

We realize room-temperature polaritons by fabricating $\lambda/2$ metallic microcavities with a spin-cast PEA_2PbI_4 active layer posessing a high degree of crystallinity resembling single crystals (Fig. S1c). By engineering the spin-coating speeds in our solution-processed cavity layers, we achieve a radial wedged cavity, with increasing cavity length from center to substrate edge which allows for facile changes to the polariton detuning (~30 meV/mm cavity mode gradient, as compared to 13 meV/mm in previously demonstrated epitaxially-grown GaAs quantum well wedged cavities[1]).

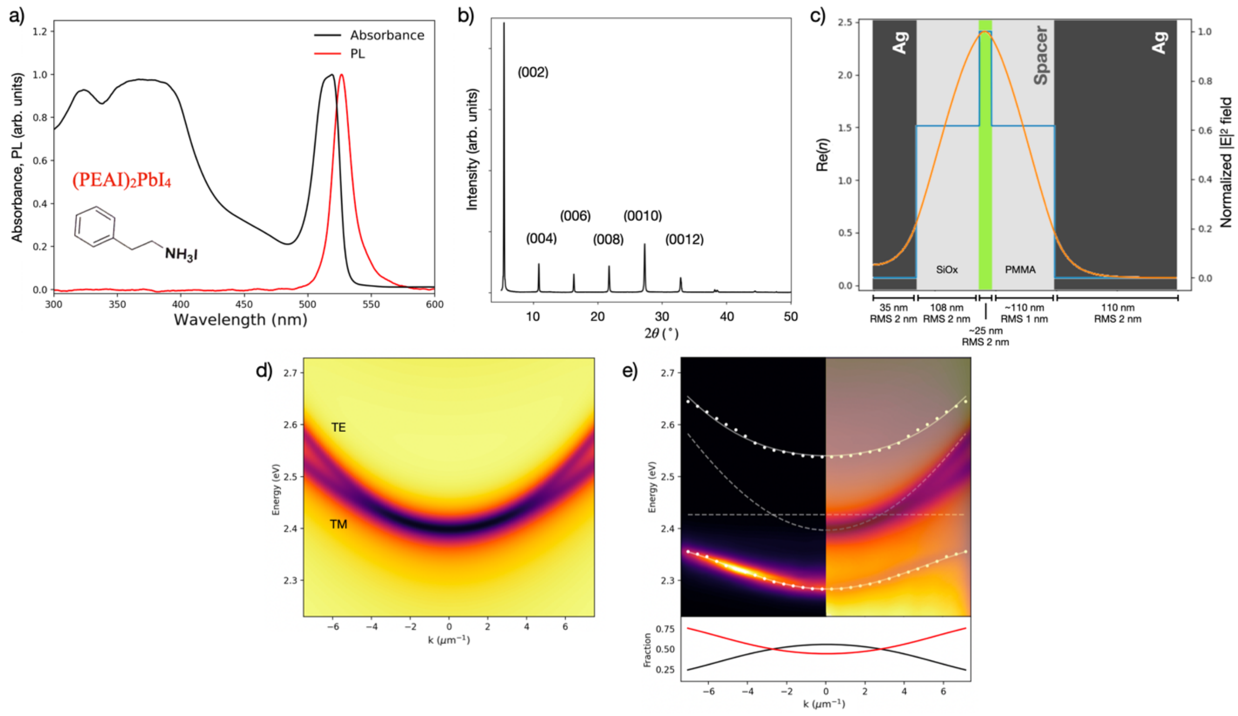

Figure S1. (a) Absorption (black trace) and photoluminescence (red trace) spectra for the PEA_2PbI_4 thin film. (b) Room-temperature XRD demonstrating a high degree of crystallinity comparable to single crystals.[2] (c) Metallic microcavity structure with normalized electric field profile (orange trace) and index of refraction (blue trace) simulated with a transfer matrix model of the cavity architecture: Ag (110nm) / SiOx (108nm) / spin-cast PEA_2PbI_4 active layer (~20nm) / PMMA (~110nm). (d) Transfer matrix model simulation of the bare $\lambda/2$ cavity (e.g., Ag / PMMA / Ag) showing the TE and TM cavity modes with normalized reflectivity intensity. The TE and TM mode splitting is zero at $k_{||}$ = 0, and increases for increasing wavevector, resulting in the splitting of polariton modes on the order of meV as confirmed by Polimeno *et al.*[6] In high-Q cavities (e.g., Q = 300,000[7]), this splitting can be more readily resolved. (e) Transfer matrix model (right) inset on Fig. S4d (fitted coupling strength of $\hbar\Omega_{Rabi}$ = 260 ± meV), with experimental upper and lower polariton branches and exciton energy. The uncoupled cavity energy is fit using main text Eq. 1, and most closely resembles the TE cavity mode of the bare cavity. Strong coupling in this system likely involves coupling to both the TE and TM modes as is seen in planar

microcavities[3]; due to the low-Q cavity in this work and the fitted uncoupled cavity mode from the strong coupling dispersion, we determine that there is primary contribution from the TE mode, similar to Zhang *et al.* in a planar perovskite microcavity.[4]

Upper and lower polariton branch extraction and fit

As shown in Fig. S2, the upper and lower polariton branches are extracted from reflectivity measurements as a function of $k_{||}$. Due to the reflectivity drop-off of the Ag top and bottom mirrors, the dip in reflectivity associated with the upper polariton branch (UPB, higher energy, lower Ag reflectivity) achieves lower contrast than the dip in reflectivity associated with the lower polariton branch (LPB, lower energy, higher Ag reflectivity). The UPB and LPB are determined by linearizing the reflectivity profile, fitting a Gaussian to the dip associated with each polariton branch, and extracting the maximum of the fitted Gaussian. The resulting branches along with the exciton energy are leveraged to determine the coupling strength (main text, Eq. 1).

On the wedged microcavity, the coupling strength is largest for the more excitonic detunings (center of the microcavity), with a slight decrease in coupling strength for more photonic detunings away from the center of the microcavity due to variation in the relative position of the 2D perovskite film in the cavity (e.g., changes to the electric field overlap within the 2D perovskite film along the top spacer layer thickness gradient) (Fig. S3). Slight variation to the coupling strength across detunings is observed, and may be attributed to variations in the perovskite thin film morphology and the error of the fit (~ +/- 5 meV). The small variation in coupling strength is ~ +/- 5 meV (Fig. S3) from detuning Δ = 0 meV, which yields an excellent fit across detunings to the theoretical UPB and LPB energies with fixed coupling strength (main text Fig. 1e, $\hbar\Omega_{Rabi}$ = 260 meV).

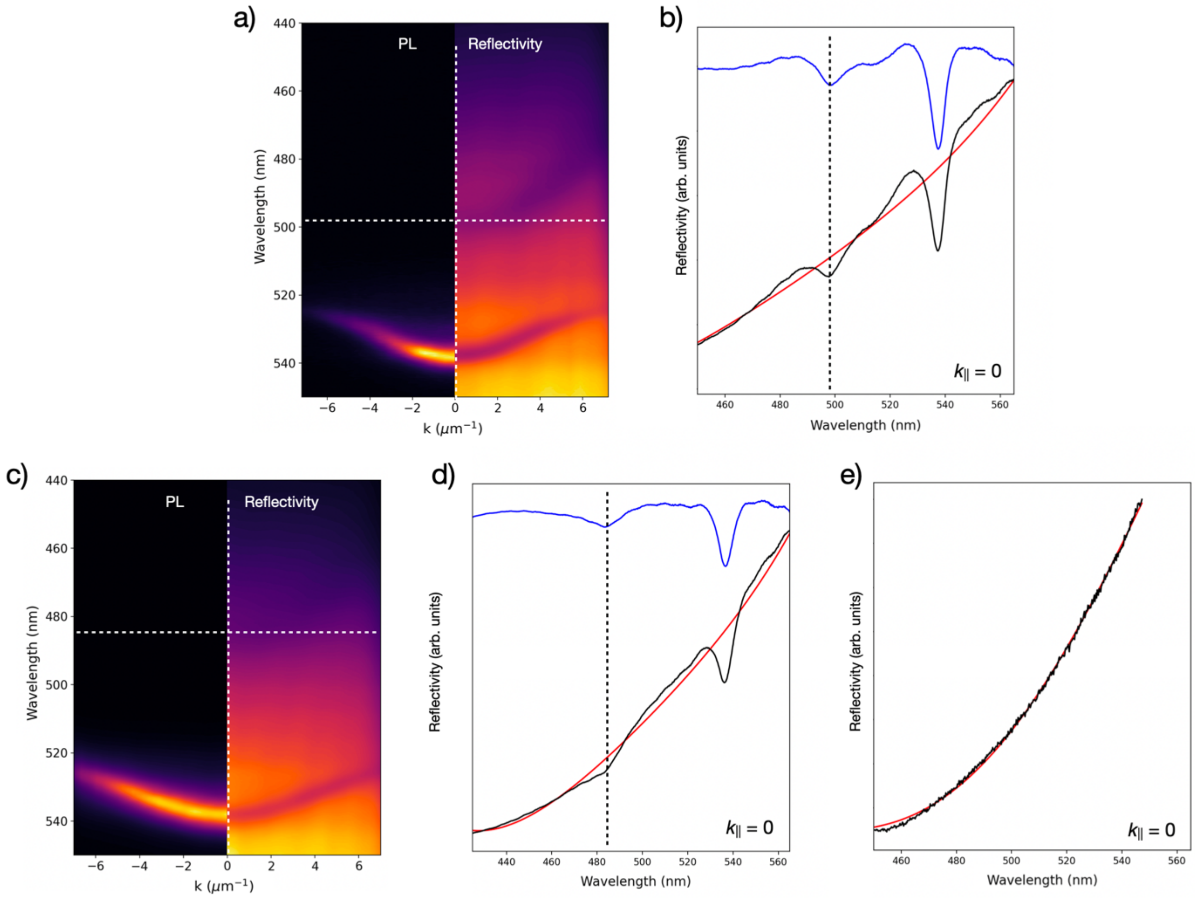

Figure S2. Strongly coupled perovskite microcavity reflectivity is shown as a function of in-plane wave vector, $k_{||}$, for two cavities with (a) lower ($\hbar\Omega_{Rabi}$ = 175 ± 5 meV) and (c) higher ($\hbar\Omega_{Rabi}$ = 260 ± 5 meV) coupling strength (white dashed traces to guide the eye, corresponding to black dashed trace). The lower polariton branch dispersion becomes flatter at high $k_{||}$ with decreasing coupling strength, as is expected for polaritons with high in-plane momentum (greater excitonic character) and lower coupling strength as observed from the Hamiltonian diagonalization and resulting energy eigenvalues.[5] (b,d) Line profiles of the reflectivity are shown for $k_{||} = 0$, with dips in reflectivity due to the upper (black dashed trace to guide the eye) and lower polariton branches. Due to the (e) reflectivity profile of the Ag metallic mirrors resulting in lower reflectivity with decreasing wavelength, the upper polariton branch dip is less pronounced than the lower polariton branch; upper and lower branches are extracted by fitting (red trace) and linearizing (blue trace) the reflectivity profile, fitting a Gaussian to each minima in the spectral regions corresponding to the upper and lower polariton branches, and taking the minimum of the fitted Gaussian at each $k_{||}$ value.

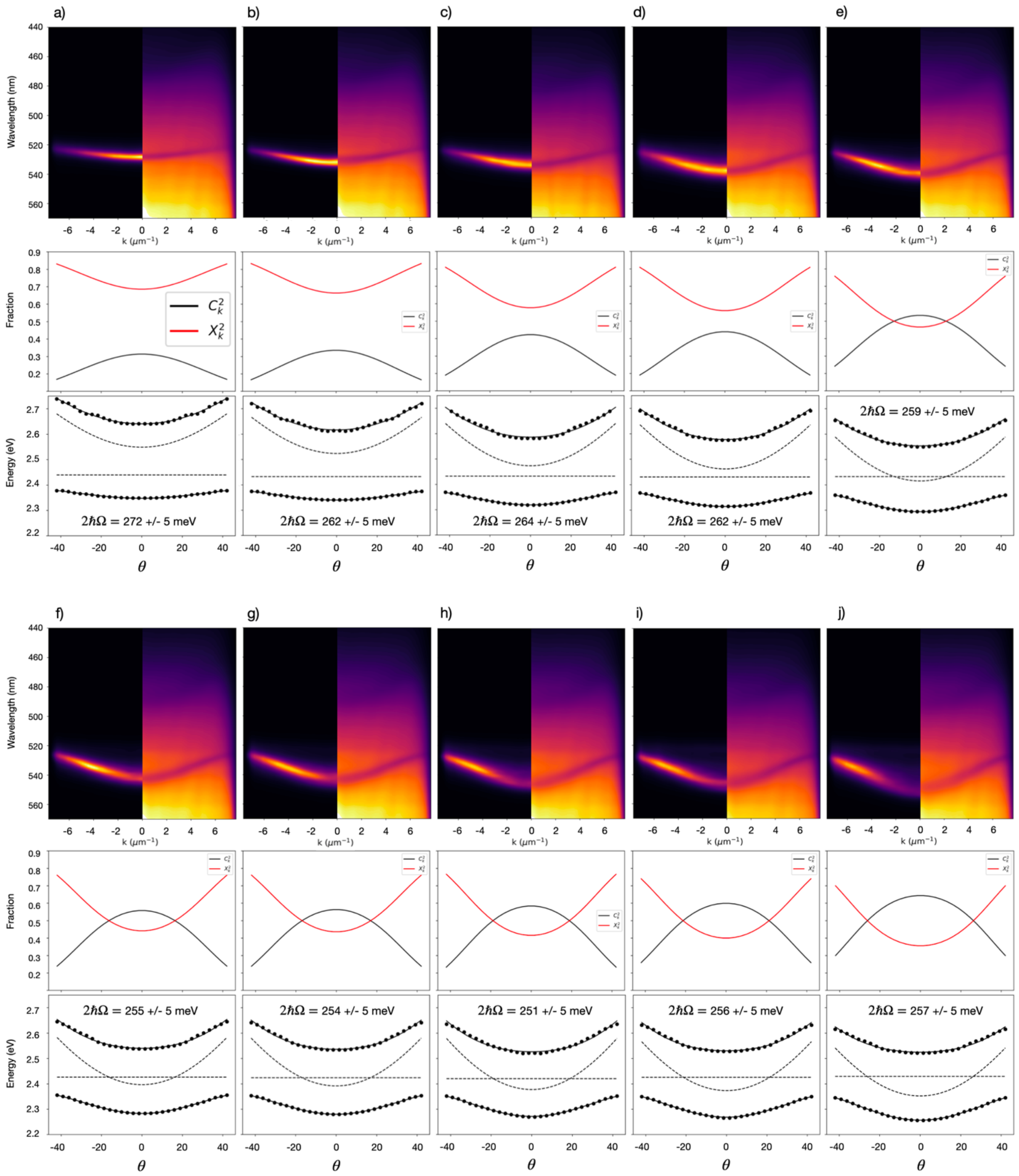

Figure S3. (a-j) Fitted UPB and LPB showing variation in Rabi splitting as a function of detuning along the cavity wedge. Several factors impact the variation in coupling strength, including the shift in the position of the active layer as a function of position in the cavity and the corresponding vairation in electric field overlap. Additionally, variations in thin film morphology and thickness will result in local regions of varying coupling strength due to either changes in the electric field overlap or number of oscillators in the active layer. As the detuning becomes more negative (away

from the microcavity center, bottom row), the coupling strength trends lower, indicating less optimal E field overlap. The variation, however, is small with respect to the coupling strenth (e.g., ~15 +/- 5 meV, or ~6% of $\hbar\Omega_{Rabi}$ = 260 meV.

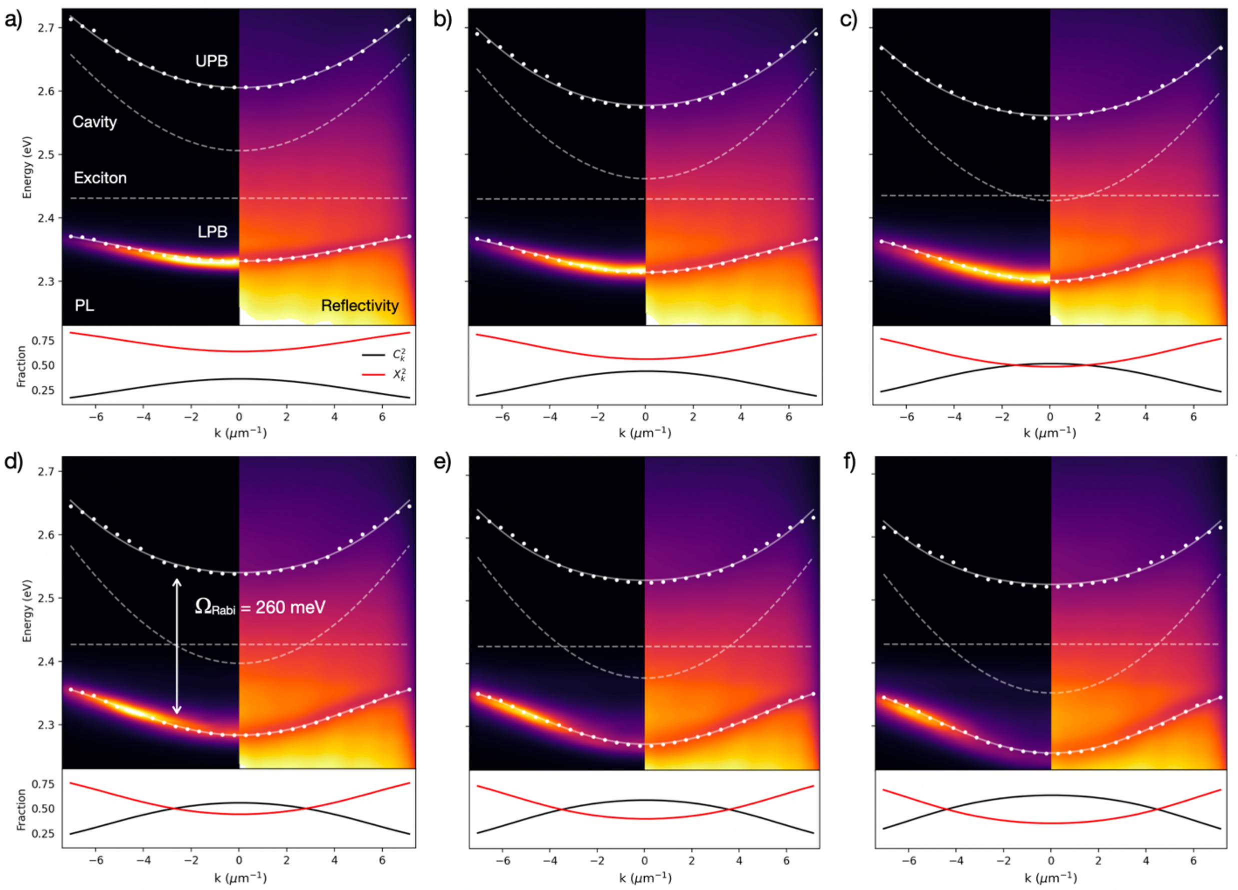

Figure S4. Exciton-polariton photoluminescence (left) and reflectivity (right) dispersions with increasing cavity length from (a) higher cavity mode energy to (f) lower cavity mode energy (f). As the cavity shifts to lower energies and the polariton dispersion becomes increasingly photonic (d-f), the bottleneck effect emerges with the greatest emission intensity at high k values. The upper and lower polariton branches are extracted from reflectivity (white dotted line) and fit (white solid line) with a Rabi splitting of $\hbar\Omega_{Rabi}$ = 260 ± 5 meV. (a-f, lower figures) Hopfield coefficients for cavity detunings (photonic fraction C_k^2, black trace; excitonic fraction X_k^2, red trace) ranging from (a) excitonic to (f) photonic depicting the light-matter characteristics of the generated polaritons as a function of $k_{//}$.

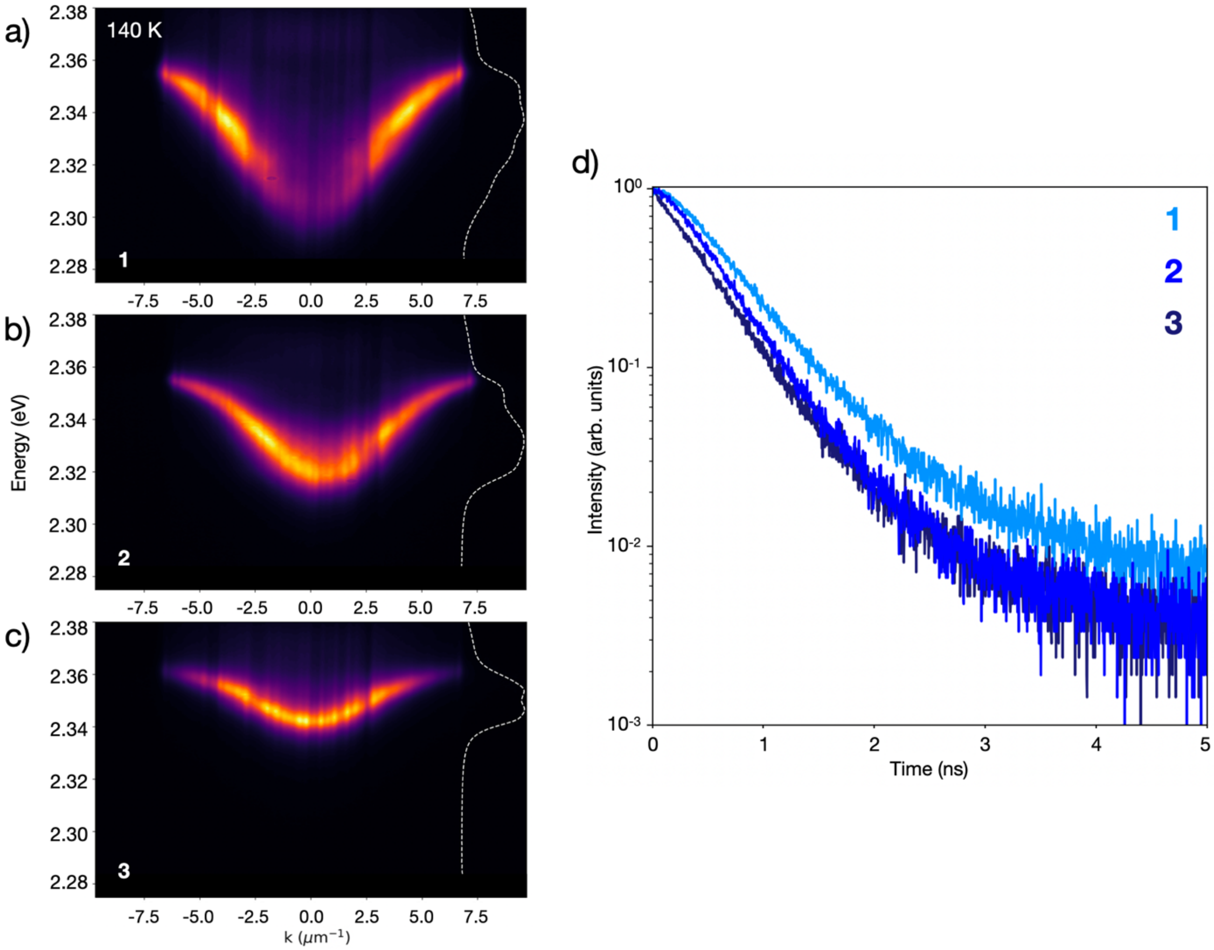

Figure S5. Photoluminesence (PL) in k-space for $\hbar\Omega_{Rabi}$ = 175 meV at 140 K for (a) highly photonic (negative) detuning with severe bottleneck, (b) photonic detuning with the beginnings of a bottleneck, and (c) excitonic (positive) detuning with no bottleneck. (d) Increasingly positive detunings result in polaritons with shorter radiative lifetimes due to the suppression of the bottleneck effect (blue traces 1-3 corresponding to (a)-(c), respectively). PL from higher $k_{||}$ in the bottleneck region corresponds to more excitonic polaritons, which possess greater scattering rates and longer radiative lifetimes, accounting for the slower emission at early timescales and longer lifetime tails.

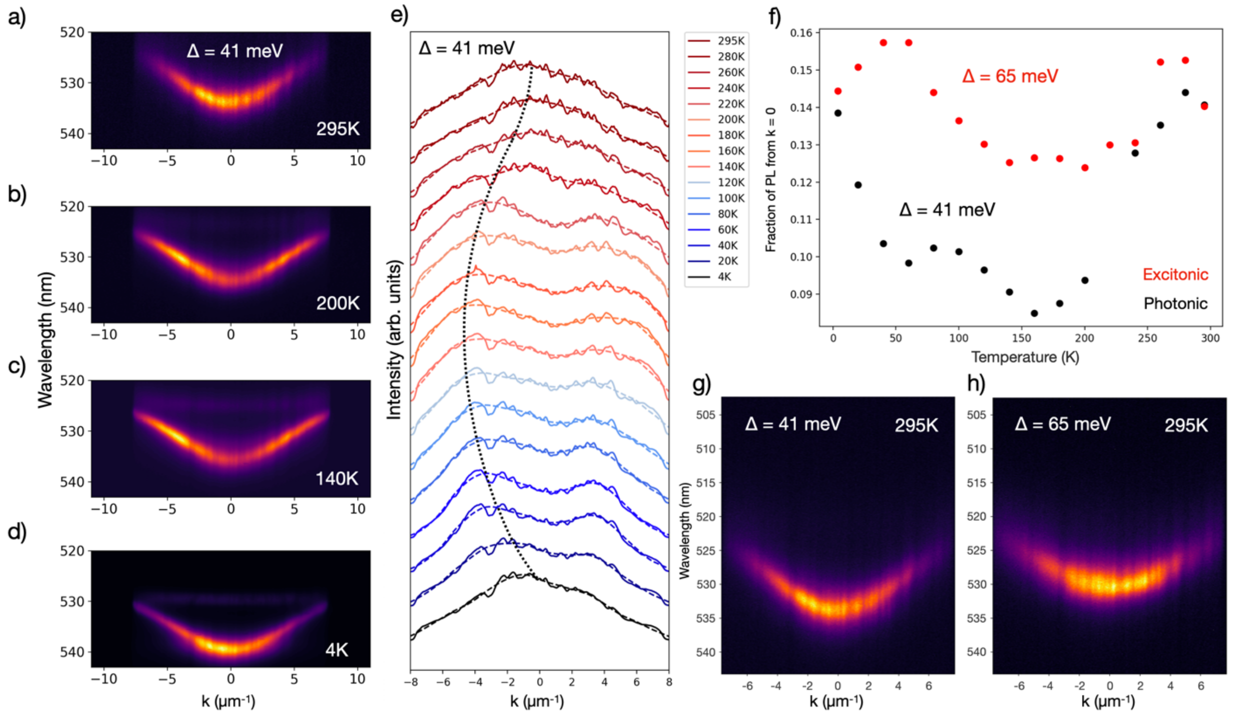

Figure S6. (a-d) Lower polariton branch (LPB) photoluminescence (PL) as a function of temperature for $\hbar\Omega_{Rabi}$ = 260 ± 5 meV revealing e) the migration of the maximum PL intensity to higher $k_{||}$ at intermediate temperatures before returning to $k_{||}$ = 0 at 4K. (e,f) The temperature-dependence of the energy-integrated PL for Δ = +41 meV showing bottlenecked PL at intermediate temperatures and emission from $k_{||}$ = 0 at sufficiently low temperatures. (g,h) PL spectra for two detunings (Δ = +41 meV and +65 meV) at 295K, corresponding to the temperature series in (f). Note: asymmetries in PL distribution arise from imperfectly flat substrate seating due to Ag cryo paste used for thermal contact. Additionally, the thicker perovskite active layer results in increased uncoupled exciton PL at elevated temperatures as compared to the thinner active layer yielding Ω_{Rabi} = 175 meV.

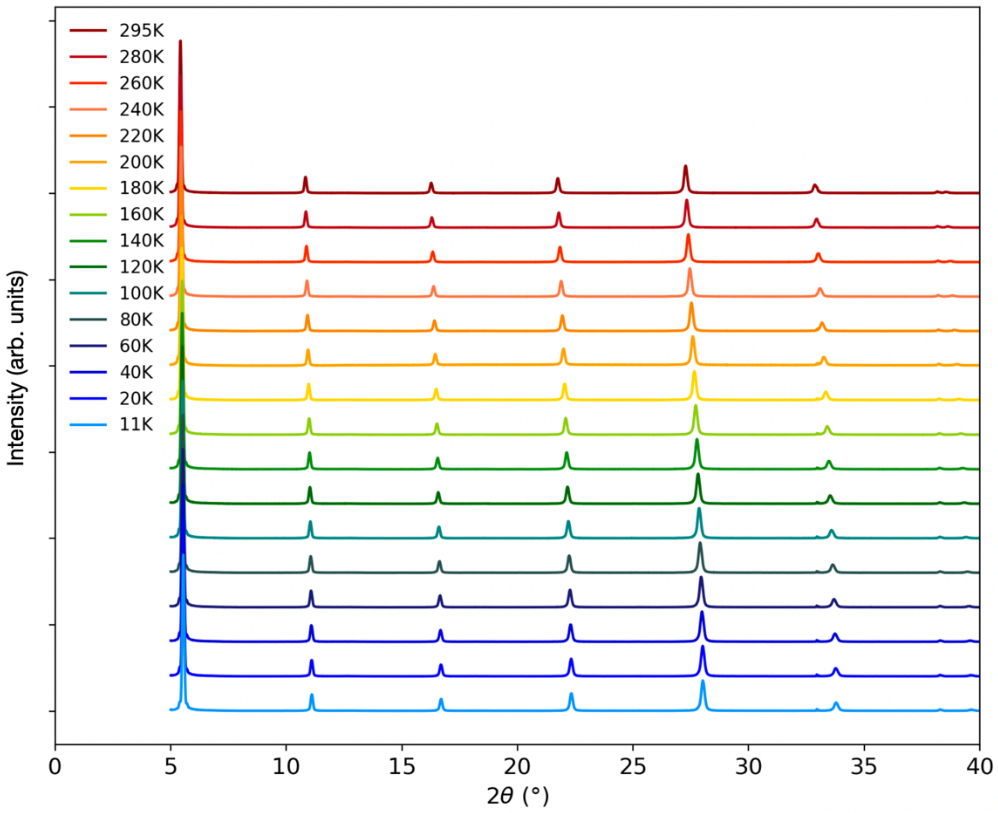

Figure S7. Temperature-dependent XRD from 295 K to 11 K showing no phase change as a function of temperature.

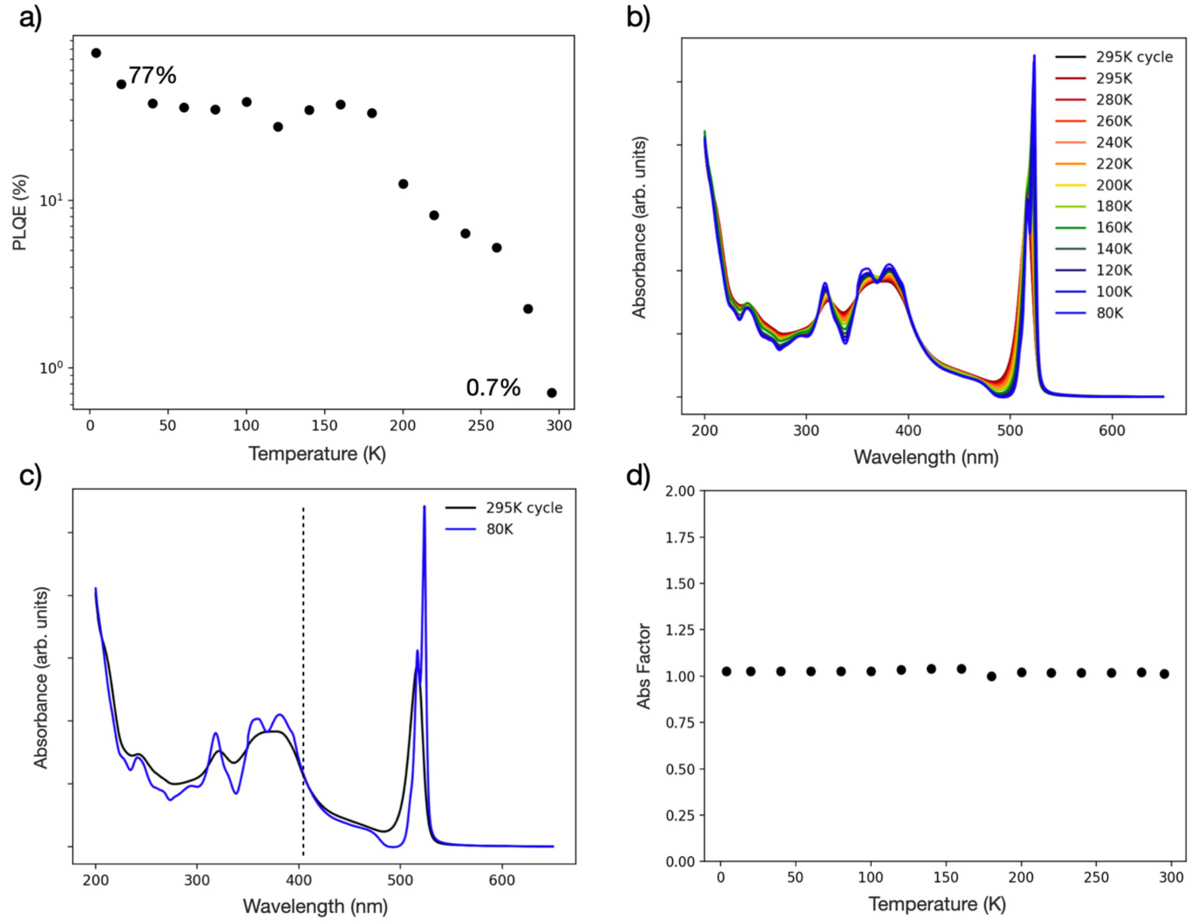

Figure S8. (a) PLQE measured at 295 K with integrating sphere and 405 nm laser excitation in cw-mode (~0.7%), and used to calculate a 100-fold PLQE increase as a function of temperature. To ensure the PL increase was not from changes in the absorption of the excitation as a function of temperature, (b) temperature-dependent absorption measurements were performed from 295 K to 80 K. The excitation wavelength was tuned to a region with very little change in absorption (c, dashed black trace indicating laser excitation wavelength), with small fluctuations in absorbance quantified in the (d) Abs Factor extrapolated to 4K.

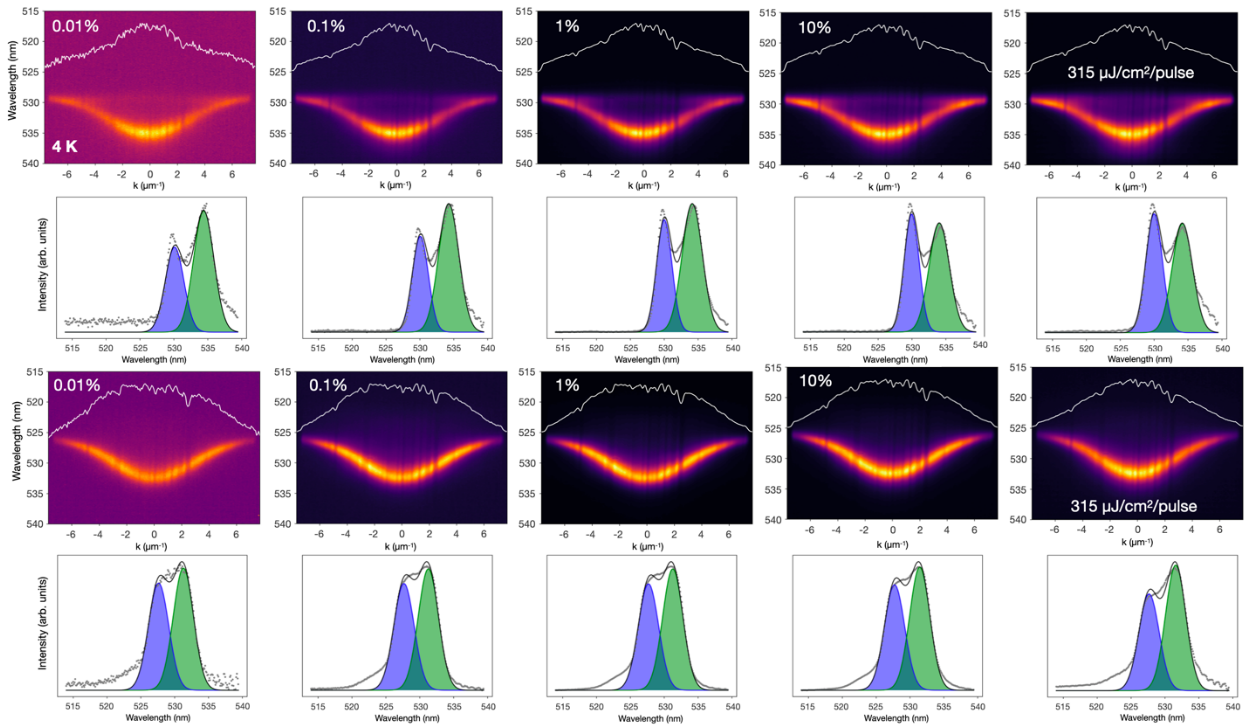

Figure S9. Lower polariton branch (LPB) photoluminescence (PL) ($\hbar\Omega_{Rabi}$ = 175 meV, Δ = +40 meV) as a function of excitation power spanning five orders of magnitude. The top row shows the *k*-space dispersion, and the bottom row shows the *k*-space-integrated PL with high-$k_{||}$ (high energy) and low- $k_{||}$ (low energy) spectral regions fit to determine whether the increase in power results in an increase in emission from the bottom of the LPB due to increased polariton-polariton scattering. No such trends are observed at 4 K, and only a weak increase in the low- $k_{||}$ region is seen at 100 K, indicating that polariton-polariton scattering is likely not the primary factor for the dramatic redistribution of PL to $k_{||}$ = 0 at low temperature. *Row 1,2*: 4 K power series (demonstrates a more rapid increase in high $k_{||}$ PL (blue Gaussian) than low $k_{||}$ (green Gaussian) indicating greater biexciton emission enhancement with increasing power); *Row 3,4:* 100 K power series (ratio between high $k_{||}$ (blue Gaussian) and low $k_{||}$ (green Gaussian) emission preserved).

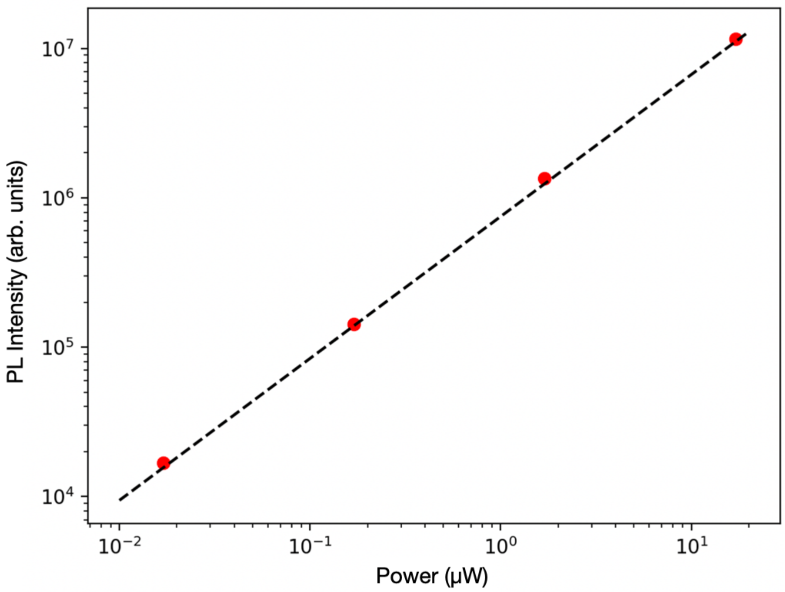

Figure S10. Integrated lower polariton branch PL at 4 K for $\hbar\Omega_{Rabi}$ = 260 meV spanning four orders of magnitude, revealing a slope of m = 0.95, consistent with bright exciton power dependence.[6–9]

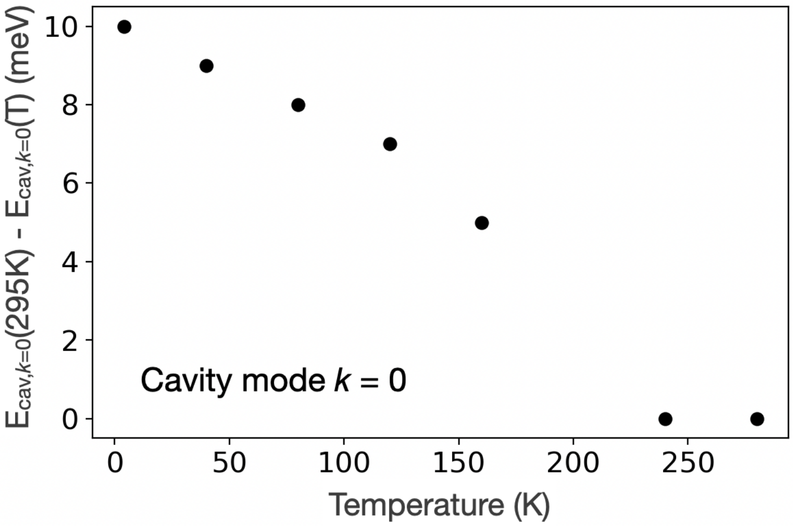

Figure S11. Cavity mode shift from 295 K (meV) at $k_{||}$ = 0 due to the thermal contraction of the microcavity as a function of temperature resulting in ~3 nm blue-shift with decreasing temperature.

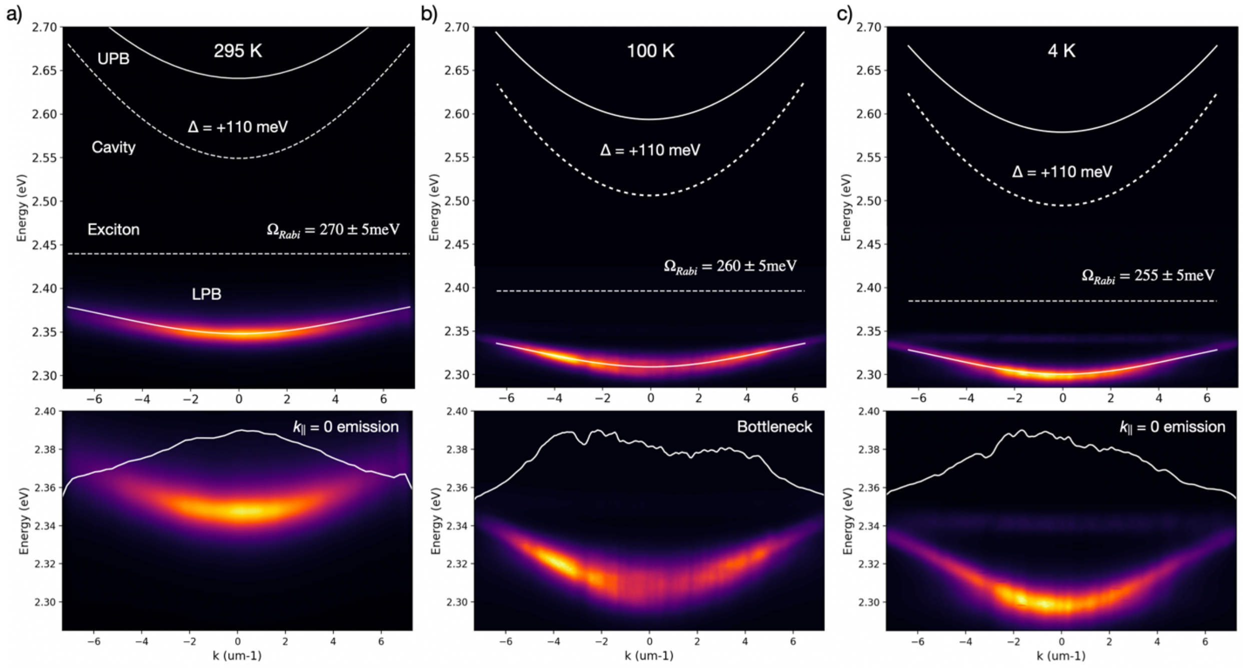

Figure S12. Lower polariton branch (LPB) emission for $\hbar\Omega_{Rabi}$ = 260 meV fixing the detuning at Δ = +110 meV by selecting a longer cavity length to keep $E_{cav} - E_{exc}$ constant as the exciton energy redshifts with decreasing temperature. Dispersions (upper panels) shown for (a) 295 K, (b) 100 K, and (c) 4 K, revealing the bottleneck effect at intermediate temperatures and emission from $k_{||} = 0$ at low temperatures (upper and lower polariton branches (solid white traces), exciton energy corresponding to the exciton absorption and bare cavity mode (dashed white traces).[10] Lower panels: LPB photoluminescence (PL) with the energy-integrated PL k-space distribution (white trace). We note that the bottleneck effect is more pronounced with increasing coupling strength (e.g., greater for $\hbar\Omega_{Rabi}$ = 260 meV than $\hbar\Omega_{Rabi}$ = 175 meV).

Low-temperature biexciton emission

In addition to the bright exciton emission visible at room temperature and the dark exciton emission visible below ~140 K, we observe a third, low energy peak emerging below ~80 K (center energy 2.305 eV at 4 K, Fig. S13). Given earlier reports, we attribute this third, low energy peak in the PL to the biexciton, for which we calculate a biexciton binding energy of $\Delta E_{XX} = 43$ meV, utilizing PL as a proxy for state energies and relative energies, in agreement with Thouin *et al*.[7] Here, the biexciton binding energy is defined as the energy difference between two free X excitons and the bound biexciton state (XX), namely $\Delta E_{XX} = 2 \cdot E_X - E_{XX}$. The first step of biexciton radiative recombination results in an emitted photon with energy $E_X - \Delta E_{XX}$, which is then followed by the emission of the remaining free exciton (X) with energy E_X. The assignment of this peak to the biexciton species is also consistent with other reports indicating a power-dependent slope of 2 and reporting a short lifetime for the lowest energy PL peak.[7,8,11]

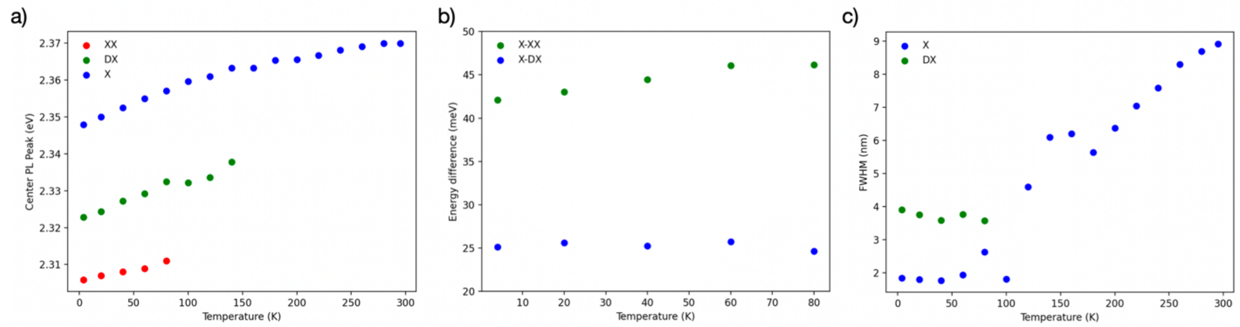

Figure S13. Bare 2D perovskite thin film energetics as a function of film temperature. (a) The center PL peak (in eV) for the bright exciton (blue), dark exciton (green), and biexciton (red). (b) The difference in PL emission energy between the bright exciton and dark exciton (X-DX, blue) and bright exciton and biexciton (X-XX, green). (c) The full-width half-maximum (FWHM, in nm) for the bright exciton (blue) and dark exciton (green, below 100 K) as a function of temperature, showing a reduction of >4x in the bright exciton FWHM.

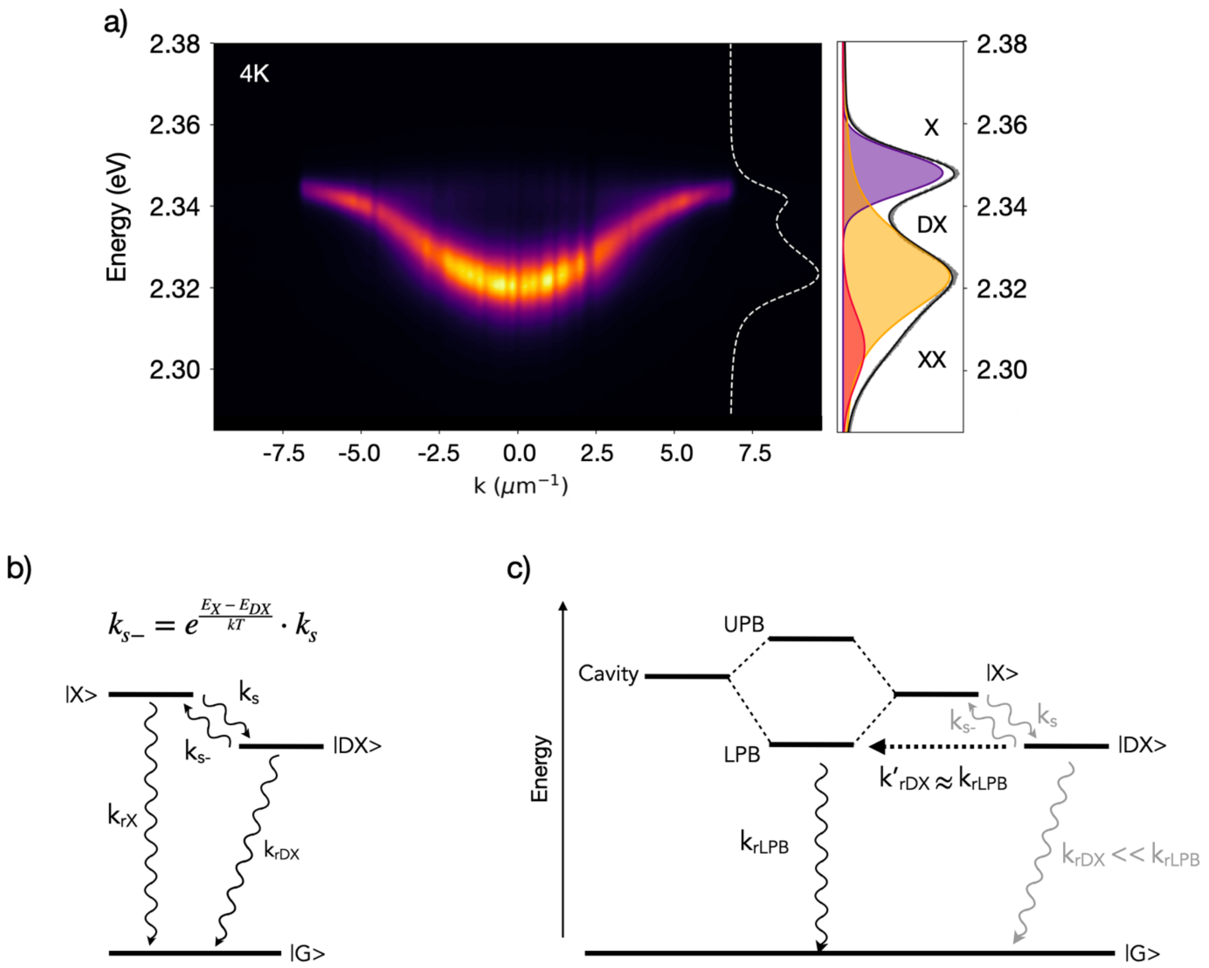

Figure S14. (a) 4 K lower polariton branch photoluminescence (PL) ($\hbar\Omega_{Rabi}$ = 175 meV, Δ = +45 meV) with primary emission from $k_{||}$ = 0. (b) 2D thin film spin-flip process from X to DX with spin-flip rate k_s. The spin-flip back to the bright state, $k_{s\text{-}}$, calculated via the Arrhenius relationship as a function of temperature (modified from ref 5).[12] (c) At low-temperature in the microcavity system when $k_{s\text{-}}$ is very slow, the strong coupling of the bright exciton to the cavity mode and resulting LPB to G polariton emission outcompetes the spin-flip process. The DX population generated immediately following photoexcitation is influenced by the isoenergetic lower polariton branch mode (dashed arrow from DX to LPB), and can directly and rapidly emit through this resonant mode, taking on the kinetics of the strongly-coupled LPB state (k'_{rDX}) which outcompetes emission from DX directly to G (k_{rDX}).

Bright/Dark Exciton Dynamics with Photon Recycling in 2D Perovskite Films

To determine the spin flip rate from the bright to dark state, a set of coupled ODEs was solved to include microscopic reversibility in Eqs. 3-5 and photon recycling. Photon recycling is the ability for a photon emitted following radiative recombination to be waveguided within the film and re-absorbed.[13] These multiple absorption/emission events serve to increase the carrier density in the

thin film for a given excitation power as compared to a material that does not exhibit photon recycling. The impact of photon recycling is dependent on the material absorption coefficient, index of refraction, radiative lifetime, and PLQE.[13,14] At low temperature, the perovskite material PLQE eventually reaches 100-fold the room temperature PLQE, indicating that photon recycling plays an ever-greater role with decreasing temperature.[14]

The probability of photon escape (P_{esc}) is calculated via Eqs. 6-7, in which the indices of refraction for the thin film, substrate, and air interface are taken into account, as well as the optical density (OD) at the wavelengths of emission.[13,15,16] For this system of a ~25 nm thick perovskite film at 4 K, the PLQE increases 100-fold to ~77%, and the OD increases by nearly a factor of two (Fig. S8, extrapolated).

$$\frac{dn_X}{dt} = -k_{rX}n_X - k_s n_X + e^{(E_X - E_{DX})/k_B T} \cdot k_s n_{DX} + \frac{c}{n_r}\sum_\lambda \alpha_\lambda \gamma_\lambda \quad (3)$$

$$\frac{dn_{DX}}{dt} = -k_{rDX}n_{DX} + k_s n_{DX} - e^{(E_X - E_{DX})/k_B T} \cdot k_s n_{DX} \quad (4)$$

$$\frac{d\gamma_\lambda}{dt} = -\frac{c}{n_r}\sum_\lambda \alpha_\lambda \gamma_\lambda + k_{rX}n_X(1 - P_{esc}) + k_{rDX}n_{DX}(1 - P_{esc}) \quad (5)$$

where k_{rX}, k_{rDX}, and k_s are the radiative recombination constant for the high energy species, low energy species, and spin-flip rate allowing for interconversion between both species, respectively, n_X and n_{DX} are the high energy and low energy carrier concentrations, respectively, E_x and E_{DX} are the energies of the bright and dark excitons, respectively, k_B is Boltzmann's constant, c is the speed of light, α_λ is the absorption coefficient at a given wavelength, γ_λ is the photon concentration within the film for a given wavelength due to radiative recombination and photon recycling, n_r is the index of refraction, and P_{esc} the probability of a radiatively recombined photon leaving the film within the escape cone.

$$\eta_t = \frac{\Omega_{esc}}{4\pi}T \approx \frac{n_{r2}^3}{n_{r1}(n_{r1}+n_{r2})^2} \quad (6)$$

$$P_{esc} = 10^{-\frac{OD_{PL}}{2}} \cdot \left(n_{t,2D-fs} + n_{t,2D-pmma} + 10^{-OD_{PL}} \cdot \left(n_{t,2D-fs} - n_{t,2D-pmma}\right)\right) \quad (7)$$

where η_t is the transmission efficiency, Ω_{esc} is the solid angle of photon escape, and n_{rx} is the index of refraction of the given material (2D perovskite/fused silica interface and 2D perovskite/PMMA interface). The transmission efficiency for both the 2D perovskite/fused silica interface and the 2D perovskite/PMMA interface is ~17%. The OD of the sister film to the microcavity active layer possessed an OD of ~0.45 at the PL emission wavelength at 4 K. Eq. 7 takes into account the various transmission efficiencies depending on the interface through which a photon escapes the film (in this instance, the interfaces have nearly the same index of refraction contrast). We estimate the probability of photon escape for these highly absorbing 2D thin films to be P_{esc} = ~20-25%.

The impact of photon recycling serves to increase the steady-state carrier density, effectively increasing the average radiative lifetime within the film as compared to a film with no photon recycling.[14] In this system at low temperature, photon recycling effectively feeds the bright state reserve, increasing the pool and allowing a portion of bright excitons to spin-flip into the dark state despite fairly slow spin-flip rates. Based on the spectral overlap between absorption and emission, it is also primarily the radiatively recombined bright excitons whose emission will be re-absorbed and recycled, creating more opportunities to engage in transfer to the energetically lowest-lying dark state at low temperature. We can quantify the number of photon recycling events in the film following the method defined by Pazos-Outón et al. to determine the impact of increasing PLQE with decreasing temperature on photon recycling events in the 2D perovskite film.[16]

$$Recycling\ events = \frac{1}{1-f_c \cdot PLQE} \tag{8}$$

$$f_c = 1 - \frac{1}{4{n_r}^2} \tag{9}$$

where f_c is the confinement factor dictated by the index of refraction, where n_r = 1.8. The dependence of the number of recycling events on PLQE is shown in Fig. S15, where, for a PLQE of ~77% (corresponding to 4 K thin film properties), the average number of recycling events is 3.5. In the radiative limit, for PLQE = 100%, the film can sustain 13 recycling events per photon.

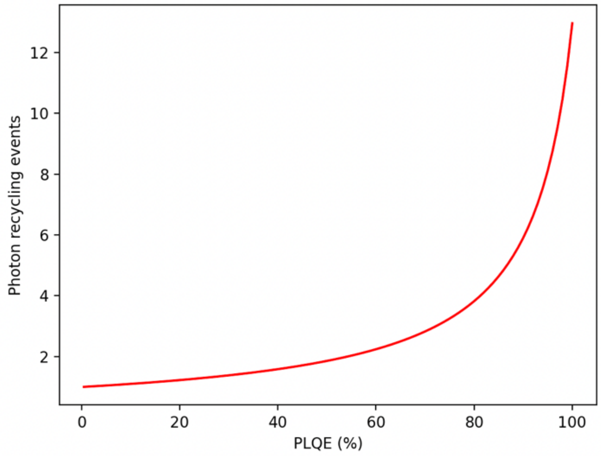

Figure S15. Number of photon recycling events in 2D perovskite thin film as a function of film PLQE, indicating that, for PLQE > ~50%, photon recycling events exceed 2 per photon and can contribute significantly to carrier dynamics.[14]

Lifetime error determination

To determine the error in the description of the coupled differential equation model to experimental data, we quantified the discrepancy via a Residual Sum of Squares (RSS) approach[17] (Eq. 10),

with the uncertainty in the lifetime calculated via the standard 95% confidence interval of the RSS for the model values as compared to experimental trace. The error of the dark exciton fast component was determined by evaluating the sensitivity of changing the fast component lifetime on the RSS of the dark exciton simulated decay and experimental decay, yielding $\tau_{60K,fast}^{DX}$ = 1.3 ± 0.05 ns (main text, Section IV). Similarly, the error of the transfer process was determined by evaluating the sensitivity of changing the transfer process lifetime on the RSS of the dark exciton simulated decay and experimental decay, yielding τ_{60K}^{s} = 0.9 ± 0.15 ns.

$$RSS = \sum_{i=1}^{n}(y_i - f(x_i))^2 \tag{10}$$

For lifetimes in which an exponential decay was fit, the same approach for determining error was utilized (e.g., 95% confidence interval of the RSS as determined by the global minimum of the exponential decay fit).

Bright/Dark Exciton Dynamics without Photon Recycling in 2D Perovsite Films

The dynamics can alternatively be simulated excluding the effects of photon recycling (Eq. 11-12). In this way, the bright exciton lifetime is required to exceed 200 ps for agreement with the raw data (Fig. S16).

$$\frac{dn_X}{dt} = -k_{rX}n_X - k_s n_X + e^{(E_X - E_{DX})/k_BT} \cdot k_s n_{DX} \tag{11}$$

$$\frac{dn_{DX}}{dt} = -k_{rDX}n_{DX} + k_s n_{DX} - e^{(E_X - E_{DX})/k_BT} \cdot k_s n_{DX} \tag{12}$$

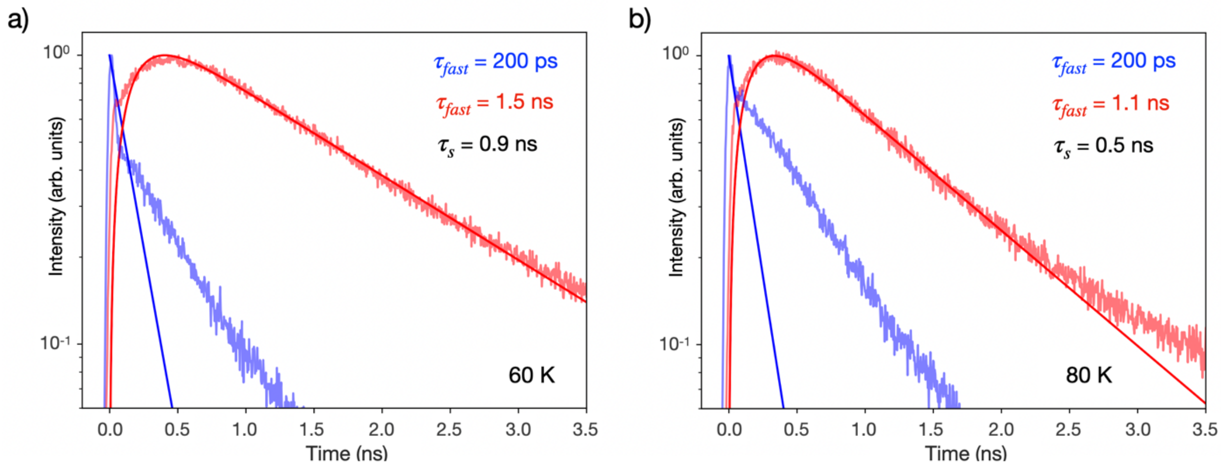

Figure S16. Lifetimes of the bright exciton (X) emission (blue) and dark exciton (DX) emission (red) in 2D perovskite films simulated with Eqs. 10-11, excluding photon recycling, at (a) 60 K (dark exciton fast emission $\tau_{60K,fast}^{DX}$ = 1.5 ± 0.05 ns; transfer process τ_{60K}^{s} = 0.9 ± 0.15 ns) and (b) 80 K (dark exciton fast emission $\tau_{80K,fast}^{DX}$ = 1.1 ± 0.2 ns; transfer process τ_{80K}^{s} = 0.5 ± 0.1 ns).

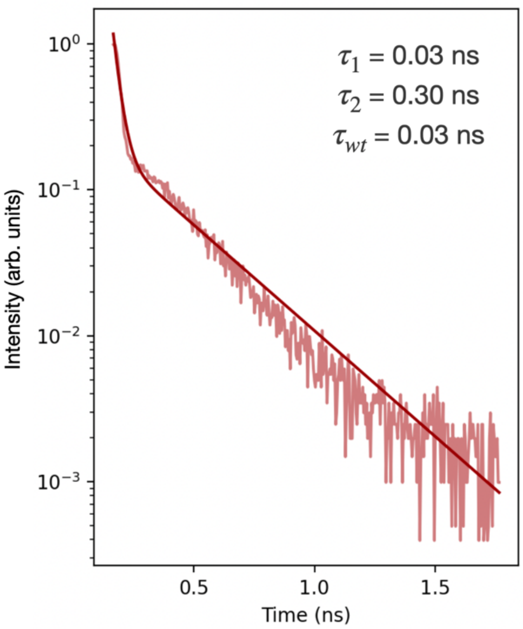

Figure S17. Instrument response function of the Toptica wavelength-tunable 80 MHz sub-ps laser and MPD detector. Fits for each trace above (τ_1 = short decay component, τ_2 = long decay component, τ_{wt} = weighted pulse duration [ns] ± 15 ps).

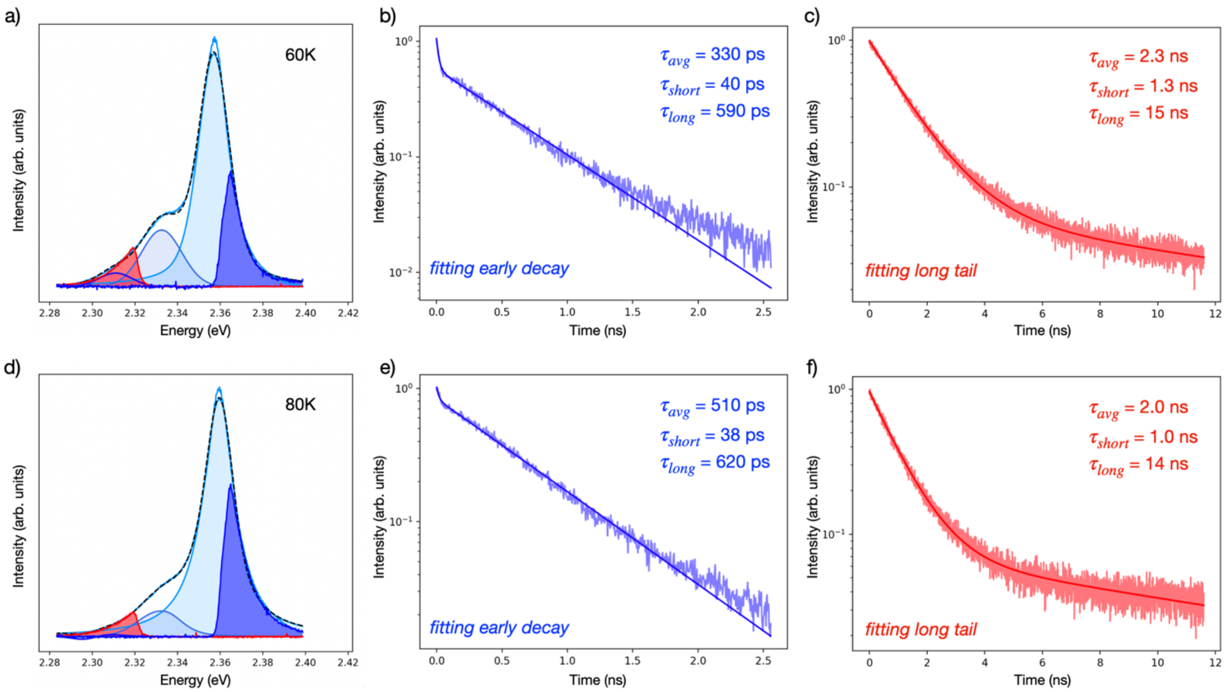

Figure S18. (a) 60 K photoluminescence (PL) spectrum of the bare film with multi-peak fitting and spectrally filtered regions highlighted in dark blue/dark red. (b) Time-resolved photoluminescence

(TRPL) trace with the tunable edge pass filter (short-pass) showing the short lifetime, fit with an exponential, attributed to the bright exciton (τ_{short} = 40 ± 20 ps) and (c) (long-pass) showing only the emisison from the dark exciton after energy transfer (truncating the delayed emission portion) to quantify the long component lifetime (τ_{short} = 1.3 ± 0.02 ns, used to inform the coupled ODE model; τ_{long} = 15 ± 3 ns). (d-f) PL spectrum at 80 K and extracted high energy short lifetime, fit with an exponential, of τ_{short} = 38 ± 20 ps. The long tail of the dark exciton decreases in lifetime at elevated temperature, with an exponential fit of τ_{long} = 14 ± 3 ns. The error in the fit of the long tail is high due to the decreased sensitivity of the residual sum of squares (RSS) within the 95% confidence interval at low signal.

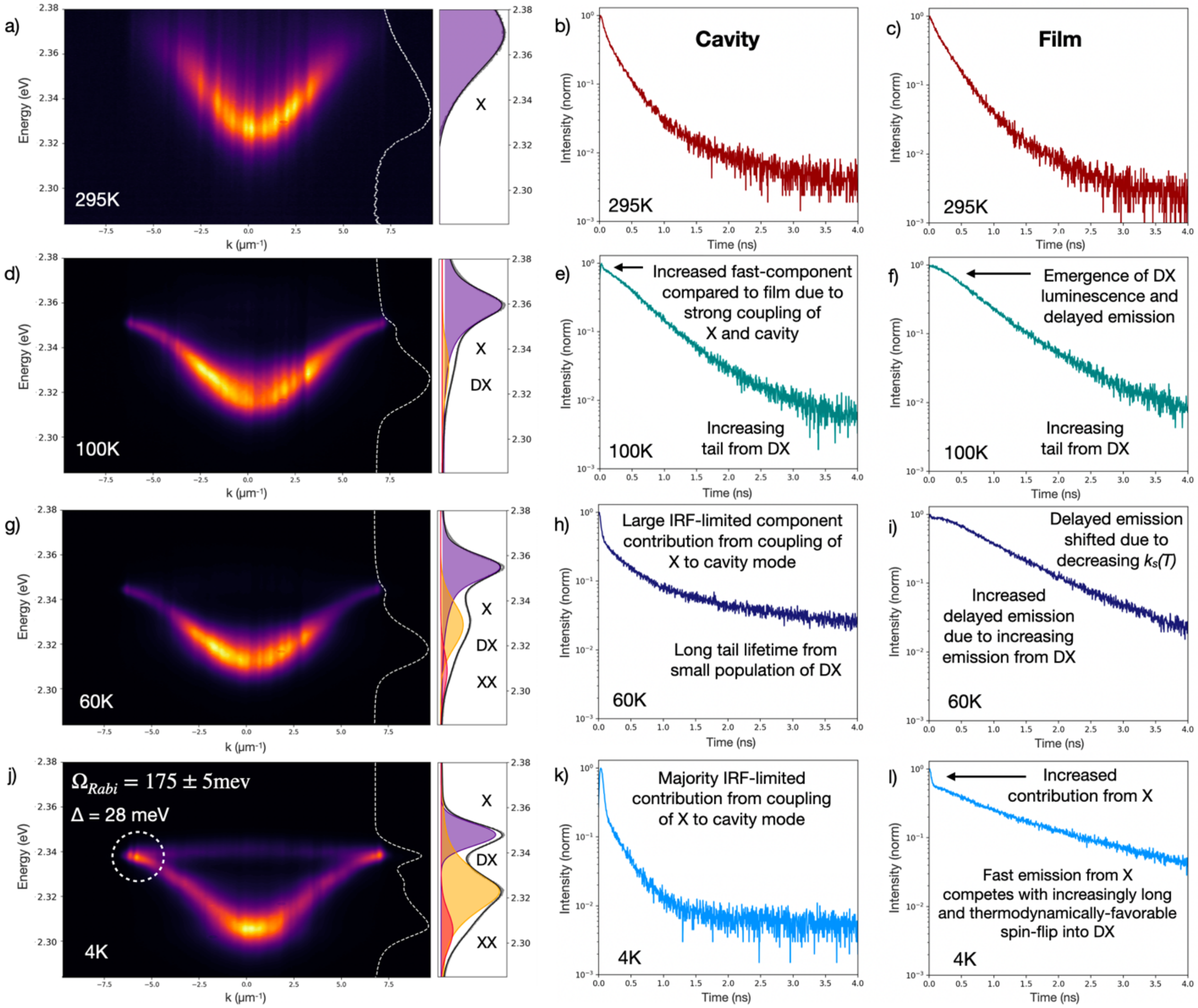

Figure S19. The lower polariton branch (LPB) emission from the microcavity ($\hbar\Omega_{Rabi}$ = 175 meV, Δ = 28 meV, right panel bare 2D film PL spectrum showing bright exciton (X), dark exciton (DX), and biexciton (XX) emission) with time-resolved photoluminescence (TRPL) decay traces for the corresponding LPB cavity emission and bare 2D film at (a,b,c) 295 K, (d,e,f) 100 K,

(g,h,i) 60 K, and (j,k,l) 4 K (white dashed circle indicating biexciton-assisted relaxation signature of high $k_{||}$ PL[10], see SI Section: Low-temperature emission from $k_{||}$ = 0: radiative pumping and biexciton-assisted relaxation). As temperature decreases, the X emission lifetime decreases and the DX emission emerges with an increasingly long lifetime, visible as a short-timescale fast component with delayed emission into a longer tail ((e,f) green trace, 100 K). In the cavity (e), the extent of delayed emission is reduced as compared to the bare 2D film (f), and the fast component contribution increased due to the additional pathway of coupling the X and cavity mode to form the strongly coupled short-lifetime polariton emissive state competing with the spin-flip from X to DX. For the bare 2D film, further reductions in temperature show (i,l) the X emission contribution increasing at early timescales as its emissive lifetime decreases and the spin-flip rate ($k_s(T)$) slows, with the DX demonstrating an increasingly long emissive lifetime and $k_{s-}(T)$ additionally slowed via the Arrhenius factor. Conversely, in the cavity, the IRF-limited strong coupling emission of X and the cavity mode competes with the spin-flip and begins to dominate the TRPL decay dynamics at 60 K (h), with weak emission contribution to the decay from the DX state resulting in a long lifetime tail. (k) At 4 K in the cavity, the delayed emission due to the slow $k_s(4K)$ and long tail from the DX is not observed, showing nearly exclusively IRF-limited strongly-coupled emission between the X and cavity mode.

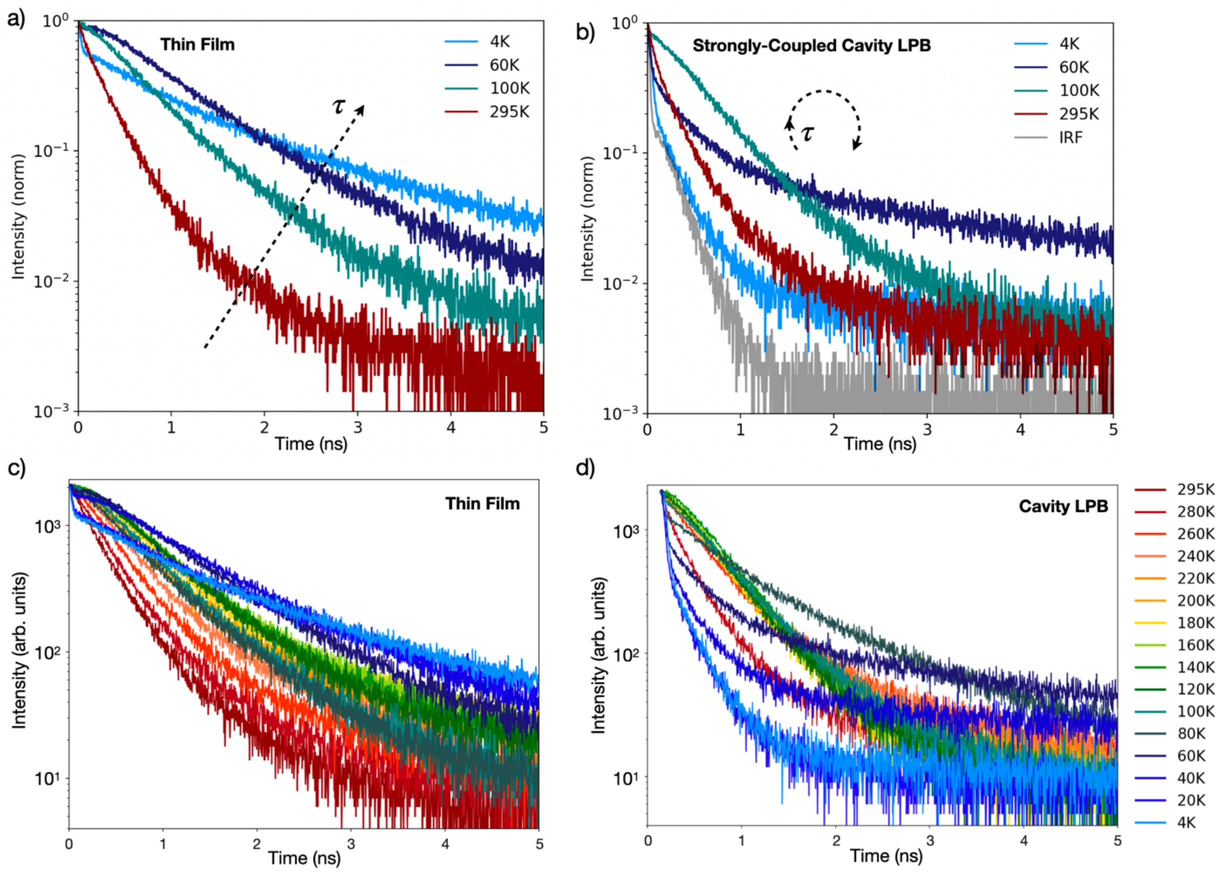

Figure S20. (a) The bare 2D perovskite film time-resolved photoluminescence decay (TRPL) traces as a function of temperature show a 295 K lifetime of $\tau_{295K} = 350$ ps, increasing with decreasing temperature. With the emergence of the dark exciton (DX) emission in the PL spectrum, the film lifetime trace develops an initial fast decay component attributed to the bright exciton (X) with a longer lifetime component attributed to the DX. (b) The strongly-coupled microcavity ($\hbar\Omega_{Rabi} = 175$ meV, $\Delta = +28$ meV) lower polariton branch (LPB) emission demonstrates similar trends to the thin film at high temperatures, but deviates sharply at low temperatures, exhibiting only the fast, IRF-limited (40 ps) lifetime of the bare 2D film initial fast decay component. (c) Temperature-dependent 2D thin film TRPL traces with finer temperature steps, showing that, with decreasing temperature, the film lifetime increases to $\tau_{180K} = 740$ ps, consistent with a reduction in non-radiative pathways which quench the lifetime. For temperatures between 180 K and 100 K, we observe reduced emission at early timescales. Below 100 K, with the prominent emergence of the DX, and subsequently XX, emission in the PL spectrum, the film lifetime trace develops an early fast decay component before the delayed emission leading into a long tail. This multi-component lifetime behavior becomes quite pronounced as the system approaches 4 K (light blue trace), and has been observed by Fang *et al.*[11] (d) The cavity LPB emission with finer temperature steps demonstrates similar trends to the thin film at high temperatures, but deviates sharply at low temperatures, exhibiting only the fast, IRF-limited (40 ps) lifetime of the bare film initial fast decay component attributed to X.

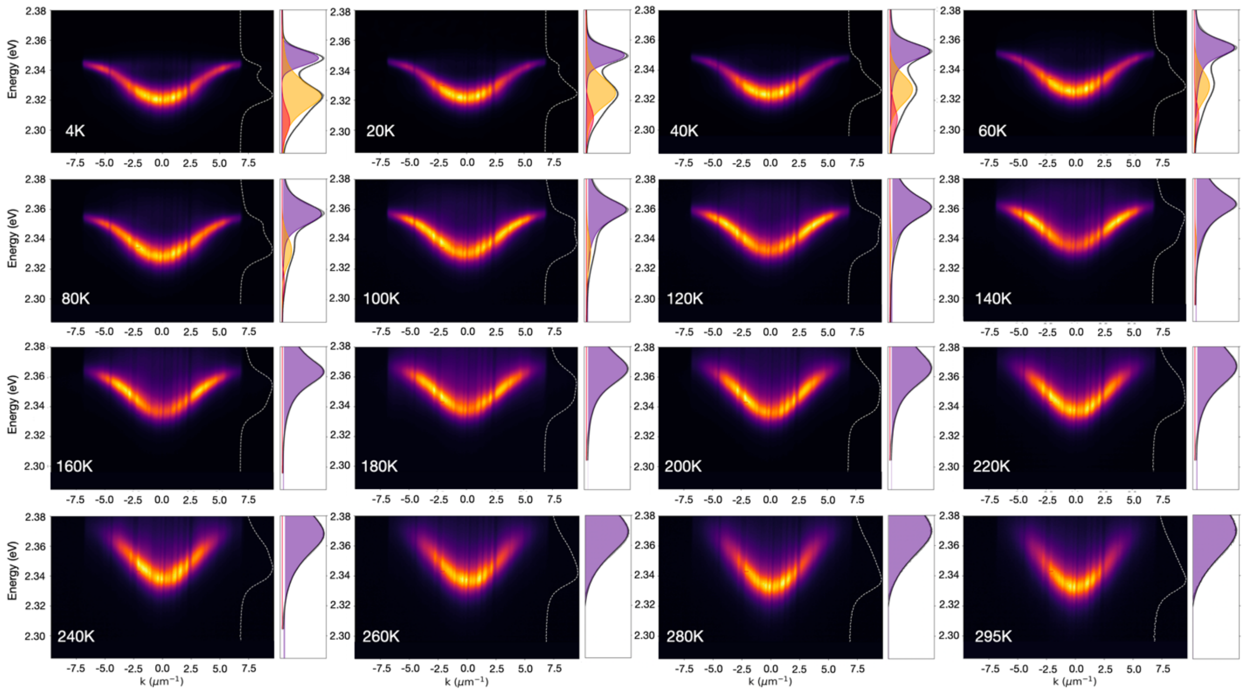

Figure S21. Photoluminescence (normalized) k-space temperature series ($\hbar\Omega_{Rabi}$ = 175 meV) from 4 K (upper left) to 295 K (lower right) for Δ = +45 meV.

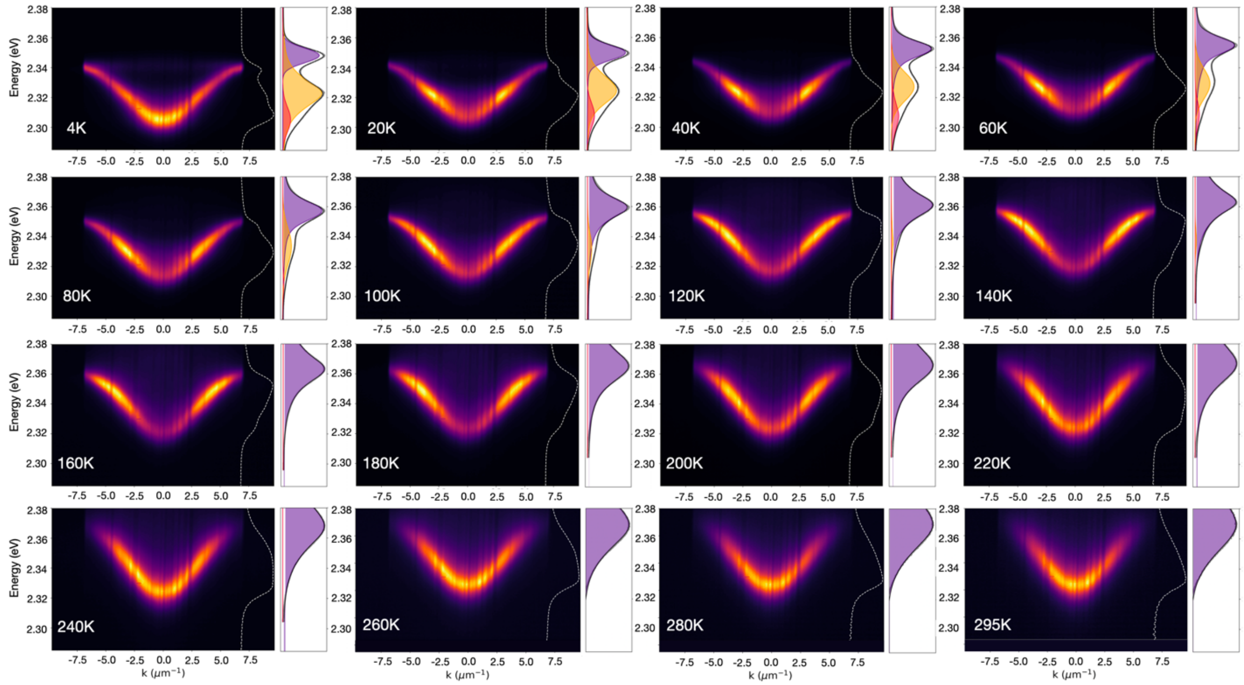

Figure S22. Photoluminescence (normalized) k-space temperature series ($\hbar\Omega_{Rabi}$ = 175 meV) from 4 K (upper left) to 295 K (lower right) for Δ = +28 meV.

Low-temperature emission from $k_{||} = 0$: radiative pumping and biexciton-assisted relaxation

In Section VI of the main text, we discuss the decay of the lower energy dark exciton (DX) through the resonant LPB mode, as has been observed in organic microcavities,[18] resulting in increased polariton density. In inorganic TMD systems, inversion of the band ordering has been observed in strongly coupled systems, with Shan *et al*. reporting a decrease in PL intensity for the TMD monolayer (WSe_2) in isolation as a function of decreasing temperature, and opposite behavior in the strong coupling regime – the brightening of the dark state only within a strongly coupled cavity due to the reversal of the energy bands enabled by tuning the lower polariton branch energy below that of the dark state.[19] Different than the behavior observed in WSe_2 by Shan *et al*., we observe the trend of increasing PL intensity for both the thin film and strongly coupled microcavity emission (Fig. S23), indicating that band inversion does not uniquely enable emission from the dark state, but rather that the dark exciton emission – increasing as a function of decreasing temperature – radiatively pumps the lower polariton branch, the dynamics and in-plane momentum of the dark state modified by the new modes resulting from strong coupling. The data in this work probing the PL spectrum and intensity as well as the spectrally filtered kinetic rates provides clear insights into the mechanism behind $k_{||} = 0$ emission at low temperature in strongly coupled PEA_2PbI_4 microcavities.

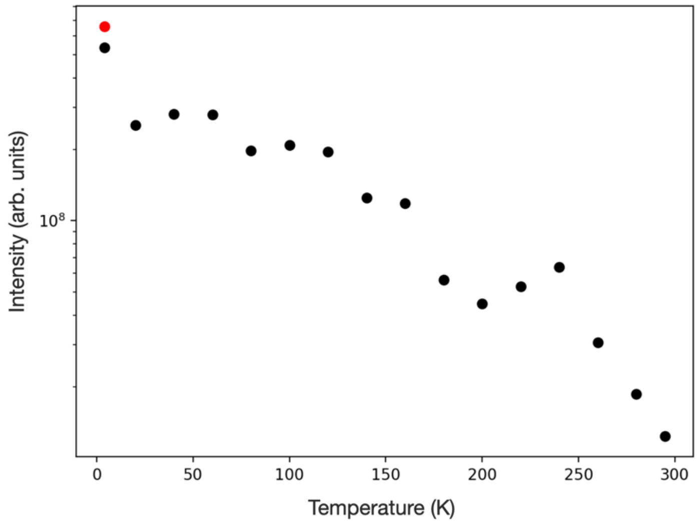

Figure S23: The strongly coupled microcavity emission ($\hbar\Omega_{Rabi}$ = 260 ± 5 meV, corresponding to Fig. S6) shows a similar increase in overall emission intensity with decreasing temperature (488 nm laser excitation) to the bare film (Fig. S8a), with slight variation as compared to (Fig. S8a) due to the small changes in absorption as a function of temperature at 488 nm. The red dot at 4 K shows a return to low temperature after the temperature cycle up to 295 K, revealing no degradation from the temperature cycling process or photodegradation after excitation.

Radiative pumping of the LPB by the biexciton state has previously been observed by Polimeno *et al*. for a PEA_2PbI_4 single crystal DBR / Ag microcavity.[20] Biexciton lasing action through the LPB was observed at a lower threshold than condensation (achieved at 1200 μJ / cm^2). Polimeno *et al.* found that the condensation threshold was achieved only when the bottom of the LPB mode was tuned to the same energy as the biexciton emission (similarly reported ~2.29 eV), with photodegradation observed before condensation was achieved when the LPB was non-resonant with the biexciton energy (e.g., detuned such that the bottom of the LPB was not isoenergetic with the biexciton state). This work supports our finding of bottleneck suppression and $k_{||} = 0$ emission when the LPB is isoenergetic with the biexciton emission, though here we importantly include the additional dynamics of bright-dark exciton transfer and radiative pumping. Together, these results suggest that harnessing such isoenergetic, radiative pumping pathways from various excitonic species may serve as efficient channels to overcoming the bottleneck effect, ultimately promoting condensation at a lower threshold.

The dark exciton and biexciton radiative pumping mechanism allows for efficient $k_{||} = 0$ emission; these pathways may be harnessed at elevated temperatures (e.g., room temperature) by the brightening of dark excitons, biexcitons, and bright excitons via chemical passivation strategies (e.g., tri-n-octylphosphine oxide-treatment to boost luminescence efficiency[21] or Mn-doping to brighten dark states by inducing large intrinsic magnetic fields[22]) and experimental strategies (e.g., applied external magnetic fields to brighten the dark states[23]).

Isoenergetic DX and XX intracavity pumping can be assisted by LO-phonon-mediated scattering and biexciton-assisted polariton relaxation for $k_{||} = 0$ emission in this 2D perovskite exciton-polariton system. These proposed scattering pathways are consistent with reports on inorganic polaritons, identifying LO-phonon-assisted transfer – including the first LO-phonon replica of the biexciton – as efficient mechanisms for directly populating $k_{||} = 0$.[10,24,25] We can observe additional signatures of biexciton-assisted relaxation given that the biexciton has two viable pathways for dissociation, as described by Corfdir *et al.*[10]:

Biexciton → LPB Polariton at $k_{||} = 0$ *+ LO-Phonon + Bright Exciton*

Biexciton → LPB Polariton at $k_{||} = 0$ *+ LO-Phonon + LPB Polariton at High* $k_{||}$

In both 4 K dispersions (main text Fig. 5e,f), there exists uncoupled exciton PL in a flat, dispersionless line at the low energy tail of the bare exciton energy band. This uncoupled PL is only visible for temperatures in which the biexciton emission is prominent (<20 K, Fig. S23). We also observe brightening of the high-$k_{||}$ wings of the LPB for T < 15 K (Fig. S23), consistent with contributions to the LPB PL distribution from both mechanisms above.

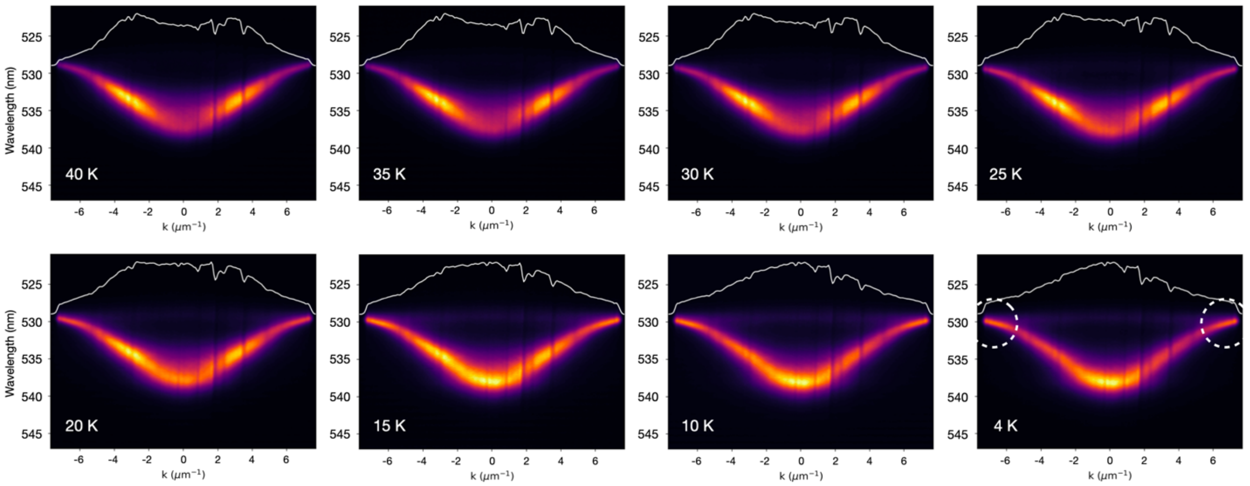

Figure S24. Photoluminescence (normalized) k-space temperature series ($\hbar\Omega_{Rabi}$ = 175 meV) from 40 K (upper left) to 4 K (lower right) for Δ = +28 meV with temperature increments of 5 K to resolve the suppression of the bottleneck and emergence of uncoupled exciton PL and high $k_{||}$ polariton PL from biexciton-assisted relaxation mechanisms.

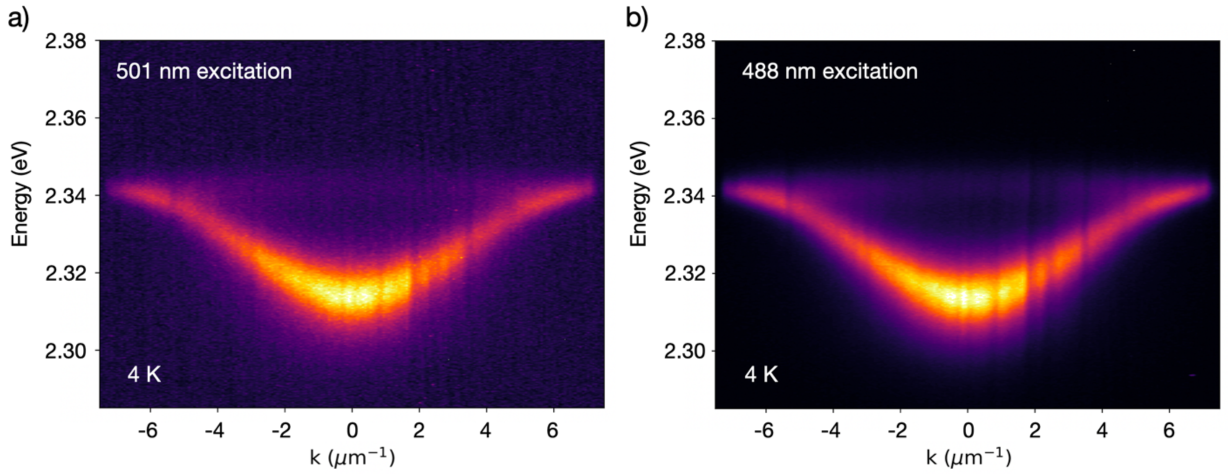

Figure S25. 4 K lower polariton branch photoluminescence (PL, normalized) ($\hbar\Omega_{Rabi}$ = 175 meV, Δ = +35 meV) with (a) 501 nm excitation and (b) 488 nm excitation showing no change to the distribution of PL in k-space as a function of excitation wavelength (e.g., via mechanisms such as resonant upper polariton branch excitation). Differences in k-space contrast stem from the decreased absorption cross section at 501 nm as compared to 488 nm.

Supplementary References

1. Steger, M., Gautham, C., Snoke, D. W., Pfeiffer, L. & West, K. Slow reflection and two-photon generation of microcavity exciton–polaritons. *Optica* **2**, 1 (2015).
2. Li, H. *et al.* Sensitive and Stable 2D Perovskite Single-Crystal X-ray Detectors Enabled by a Supramolecular Anchor. *Advanced Materials* **32**, 2003790 (2020).
3. Spencer, M. S. *et al.* Spin-orbit–coupled exciton-polariton condensates in lead halide perovskites. *Sci Adv* **7**, (2021).
4. Zhang, S. *et al.* Trapped Exciton–Polariton Condensate by Spatial Confinement in a Perovskite Microcavity. *ACS Photonics* **7**, 327–337 (2020).
5. Deng, H., Haug, H. & Yamamoto, Y. Exciton-polariton Bose-Einstein condensation. *Rev Mod Phys* **82**, 1489–1537 (2010).
6. Kondo, T., Azuma, T., Yuasa, T. & Ito, R. Biexciton lasing in the layered perovskite-type material (C6H13NH3)2PbI4. *Solid State Commun* **105**, (1998).
7. Thouin, F. *et al.* Stable biexcitons in two-dimensional metal-halide perovskites with strong dynamic lattice disorder. *Phys Rev Mater* **2**, 034001 (2018).
8. Tang, W. *et al.* Thickness dependent dark exciton emission in (PEA)2PbI4 nanoflake and its brightening by in-plane magnetic field. *ArXiv* (2021).
9. Kim, J. C., Wake, D. R. & Wolfe, J. P. Thermodynamics of biexcitons in a GaAs quantum well. *Phys Rev B* **50**, 15099–15107 (1994).
10. Corfdir, P. *et al.* Impact of biexcitons on the relaxation mechanisms of polaritons in III-nitride based multiple quantum well microcavities. *Phys Rev B* **85**, 245308 (2012).
11. Fang, H. *et al.* Band-Edge Exciton Fine Structure and Exciton Recombination Dynamics in Single Crystals of Layered Hybrid Perovskites. *Adv Funct Mater* **30**, 1907979 (2020).
12. Bawendi, M. G., Carroll, P. J., Wilson, W. L. & Brus, L. E. Luminescence properties of CdSe quantum crystallites: Resonance between interior and surface localized states. *J Chem Phys* **96**, 946–954 (1992).
13. Brenes, R., Laitz, M., Jean, J., deQuilettes, D. W. & Bulović, V. Benefit from Photon Recycling at the Maximum-Power Point of State-of-the-Art Perovskite Solar Cells. *Phys Rev Appl* **12**, 014017 (2019).
14. deQuilettes, D. W. *et al.* Impact of Photon Recycling, Grain Boundaries, and Nonlinear Recombination on Energy Transport in Semiconductors. *ACS Photonics* **9**, 110–122 (2022).
15. Richter, J. M. *et al.* Enhancing photoluminescence yields in lead halide perovskites by photon recycling and light out-coupling. *Nat Commun* **7**, 13941 (2016).
16. Pazos-Outon, L. M. *et al.* Photon recycling in lead iodide perovskite solar cells. *Science (1979)* **351**, 1430–1433 (2016).
17. Hartono, N. T. P. *et al.* How machine learning can help select capping layers to suppress perovskite degradation. *Nat Commun* **11**, 4172 (2020).
18. Dhavamani, A., Haeberlé, L., Wang, J., Kéna-Cohen, S. & Arnold, M. S. Cavity-Mediated Hybridization of Bright and Dark Excitons in an Ultrastrongly Coupled Carbon Nanotube Microcavity. *ACS Photonics* **8**, 2375–2383 (2021).
19. Shan, H. *et al.* Brightening of a dark monolayer semiconductor via strong light-matter coupling in a cavity. *Nat Commun* **13**, 3001 (2022).
20. Polimeno, L. *et al.* Observation of Two Thresholds Leading to Polariton Condensation in 2D Hybrid Perovskites. *Adv Opt Mater* **8**, 2000176 (2020).

21. Dequilettes, D. W. *et al.* Photoluminescence Lifetimes Exceeding 8 μs and Quantum Yields Exceeding 30% in Hybrid Perovskite Thin Films by Ligand Passivation. *ACS Energy Lett* **1**, 438–444 (2016).
22. Lohmann, S., Cai, T., Morrow, D. J., Chen, O. & Ma, X. Brightening of Dark States in CsPbBr3 Quantum Dots Caused by Light-Induced Magnetism. *Small* **17**, 2101527 (2021).
23. Wang, S. *et al.* Thickness-Dependent Dark-Bright Exciton Splitting and Phonon Bottleneck in CsPbBr3-Based Nanoplatelets Revealed via Magneto-Optical Spectroscopy. *Nano Lett* **22**, 7011–7019 (2022).
24. Maragkou, M., Grundy, A. J. D., Ostatnický, T. & Lagoudakis, P. G. Longitudinal optical phonon assisted polariton laser. *Appl Phys Lett* **97**, 111110 (2010).
25. Bœuf, F. *et al.* Evidence of Polariton Stimulation in Semiconductor Microcavities. *physica status solidi (a)* **183**, 29–33 (2001).
